# Forces Shaping the Fastest Evolving Regions in the Human Genome

**DOI:** 10.1371/journal.pgen.0020168

**Published:** 2006-10-13

**Authors:** Katherine S Pollard, Sofie R Salama, Bryan King, Andrew D Kern, Tim Dreszer, Sol Katzman, Adam Siepel, Jakob S Pedersen, Gill Bejerano, Robert Baertsch, Kate R Rosenbloom, Jim Kent, David Haussler

**Affiliations:** 1 Department of Biomolecular Engineering, University of California Santa Cruz, Santa Cruz, California, United States of America; 2 Howard Hughes Medical Institute, University of California Santa Cruz, Santa Cruz, California, United States of America; University of Chicago, United States of America

## Abstract

Comparative genomics allow us to search the human genome for segments that were extensively changed in the last ~5 million years since divergence from our common ancestor with chimpanzee, but are highly conserved in other species and thus are likely to be functional. We found 202 genomic elements that are highly conserved in vertebrates but show evidence of significantly accelerated substitution rates in human. These are mostly in non-coding DNA, often near genes associated with transcription and DNA binding. Resequencing confirmed that the five most accelerated elements are dramatically changed in human but not in other primates, with seven times more substitutions in human than in chimp. The accelerated elements, and in particular the top five, show a strong bias for adenine and thymine to guanine and cytosine nucleotide changes and are disproportionately located in high recombination and high guanine and cytosine content environments near telomeres, suggesting either biased gene conversion or isochore selection. In addition, there is some evidence of directional selection in the regions containing the two most accelerated regions. A combination of evolutionary forces has contributed to accelerated evolution of the fastest evolving elements in the human genome.

## Synopsis

Studies of differences between the chimpanzee and human genomes have focused on protein-coding genes. However, examples of amino acid changes between chimp and human have not been able to explain most of the phenotypic differences between us and our fellow hominoids. King and Wilson (1975) proposed that the main differences between chimps and humans will be found in non-coding regulatory DNA. Consistent with this hypothesis, recent whole-genome scans for evolutionarily conserved DNA elements that have evolved rapidly since our divergence from the chimp-human ancestor have discovered largely non-coding regions. The authors investigate a carefully screened set of 202 such human accelerated regions (HARs). Most of these HARs do not code for proteins, but instead are located in introns and intergenic regions near protein-coding genes. The set of genes near HARs is enriched for transcription factors, suggesting that the HARs may play important roles in gene regulation. This study also discovers a striking adenine and thymine to guanine and cytosine bias among the human-specific changes in HARs. This suggests the involvement of biased gene conversion or a selective force to increase guanine and cytosine content. Some HARs may also have been under positive selection. Hence, there is likely more than one evolutionary force shaping the fastest evolving regions of the human genome.

## Introduction

The chimpanzees are our closest relatives in the tree of life. Recent sequencing and assembly of the genome of the common chimp *(Pan troglodytes)* offers an unprecedented opportunity to understand primate evolution and to identify those changes in the ancestral hominoid genome which gave rise to the modern human species [1]. Primate genome comparisons are expected to shed light on questions as diverse as the origins of speech [2,3] and the progression of HIV infection to AIDS [4]. Whereas the aim of comparative studies of human and rodent genomes [5,6] is typically to identify genomic elements that are evolutionarily conserved (and therefore presumably functionally important given the ~150 million years of evolution separating the species), we look to the chimpanzee genome to better understand what is uniquely human about our genome. One goal is to find DNA elements that show evidence of rapid evolution in the human lineage, where “accelerated” or “rapid” refers to a general increase in the rate of nucleotide substitution. Pollard et al. [7] used comparative genomics to identify 49 such human accelerated regions (HARs) that are evolving very slowly in vertebrates but have changed significantly in the human lineage. The most accelerated of these, HAR1, was found to be a novel RNA gene expressed during neocortical development [7]. In this paper, we investigate the properties of a larger set of 202 carefully screened HARs in order to unravel the evolutionary forces at work behind the fastest evolving regions of the human genome.

To address questions of human-specific molecular evolution it is not sufficient to simply identify all nucleotide differences between the human and chimpanzee genomes. Despite being a small fraction of the human genome, the number of human bases that differ from the corresponding chimp base is still large (nearly 29 million bases), and it is likely that most of these differences do not have a functional consequence. Furthermore, many authors, starting with the seminal work of King and Wilson [8], have suggested that the majority of the changes that distinguish humans from other hominoids will be found in the 98.5% of the genome that is non-coding DNA, which is a vast territory to search. To identify changes that may be functional, we focus on the set of regions of the human genome of at least 100 base pairs (bp) that appear to have been under strong negative selection up to the common ancestor of human and chimp (as evidenced by high sequence identity between chimp and rodents), but exhibit a cluster of changes in human compared to chimp. Our expectation is that the selective constraint on the most extremely accelerated regions of the human genome may have switched from negative to positive (and possibly back to negative) some time in the last 5−6 million years.

Fast-evolving genomic elements, primarily protein coding, have been reported previously. Some well-known examples include genes involved in immunity and reproduction, as well as hypervariable regions of mitochondrial DNA (d-loop, HV1–3) and somatic mutation of antibody variable regions. Recent publications have documented accelerated evolution of nervous system genes in primates (particularly in the ape/human lineage) compared to rodents [9] and of genes involved in spermatogenesis, sensory perception, and immune defenses in human compared to chimp [10]. Numerous statistical tests of selection in protein coding regions have been used in these studies [11].

One population genetic test for selection that is applicable to non-coding DNA is the Hudson-Kreitman-Aguadé (HKA) test [12], which is based on the ratio of within-species polymorphism to between-species divergence. Another is Fay and Wu's *H* statistic, which was employed by Rockman et al*.* to detect positive selection in the regulatory region of *PDYN,* an endogenous opiate receptor ligand [13]. One molecular evolutionary test that uses non-coding sequence is the approach proposed by Wong and Nielsen to detect positive selection in the non-coding regions of a gene using both its coding and non-coding sequence [14]. The motivating idea behind these and many other studies is that departures from the neutral model of molecular evolution are likely to indicate natural selection. Such tests are designed to find regions with patterns of polymorphism or substitution which do not match neutral expectations. Our strategy, in contrast, is to first detect *any* increase in the rate of nucleotide substitutions on the human lineage and then apply a separate analysis to distinguish positive selection from relaxation of negative selection.

Positive selection and relaxation of negative selection are not the only evolutionary forces that result in accelerated nucleotide evolution. It is well known that population subdivision and changes in population size can lead to the rapid fixation of segregating alleles [11]. Mutation rate variation can also cause genomic regions to have different substitution rates without any change in fixation rate. Recent studies of guanine and cytosine (GC)-isochores in the mammalian genome have suggested the importance of another selectively neutral evolutionary process that affects nucleotide evolution. As described in the work of Laurent Duret and others [15−17], biased gene conversion (BGC) is a mechanism caused by the mutagenic effects of recombination [18] combined with the preference in recombination-associated DNA repair towards strong (GC) versus weak (adenine and thymine [AT]) nucleotide pairs at non-Watson-Crick heterozygous sites in heteroduplex DNA during crossover in meiosis. Thus, beginning with random mutations, BGC results in an increased probability of fixation of G and C alleles. The tests for positive selection discussed above look for evidence against the neutral model and therefore cannot distinguish positive selection from BGC or various demographic explanations. Nor can they confirm or refute the original selectionist model of the evolution of GC-isochores put forth by Bernardi and colleagues [19,20]. Recent data also show that increasing the GC content of transcribed sequences increases their exression level, which can have high adaptive value [21]. Some effort has been made to characterize the different signatures of these evolutionary forces. Polymorphism data, for example, can be used to distinguish between mutation bias and fixation bias [15−17]. In this study, we use the presence and extent of selective sweeps and substitution bias in HARs to try to tease these effects apart.

Essential to our exploration of these accelerated regions is an accurate assessment of substitution rate variation. Many methods have been developed to examine the “molecular clock” hypothesis (roughly equal rates of molecular evolution across lineages) [22]; these employ relative rates [23], likelihood ratios [24,25], chi-square statistics [26], or the index of dispersion [27]. Likelihood ratio tests (LRT) are popular because they sum over all possible ancestral sequences and therefore account directly for uncertainty in the number of substitutions in different lineages. A possible disadvantage of LRT methods in this setting, however, is that they require fitting a molecular evolutionary model to each region of interest, using as little as 100 bp of sequence. To address this issue, we begin with a genome-wide model for conserved regions and fit only one additional parameter, a scale factor that represents a faster or slower substitution rate across the whole tree, to obtain a null model with genome-wide average relative rates in all lineages for each genomic region. Then, a model with a second additional parameter for acceleration on the human branch is fit for each region. The LRT statistic for a region is the ratio of the likelihood of the model with acceleration on the human branch to the model without human acceleration. The significance of the LRT statistics can be assessed by simulation from the genome-wide null model. Another potential problem with the LRT is that the data used to test for acceleration (a multiple species alignment) are not independent from the data used to select the regions of interest, since the chimpanzee, mouse, and rat genomes are used in each step. To circumvent this dependence and to provide an unbiased assessment of significance, we exclude the branches of the phylogeny that were used to identify regions when we perform the LRT. This approach has been validated by comparison with other commonly used and novel methods [7].

Using this LRT method, we discovered 202 HARs with significantly elevated substitution rates in the human lineage. This set has been extensively screened for assembly and alignment errors, including removing examples of pseudogenes and misaligned paralogs, producing a high confidence, ranked list of accelerated elements. Of these regions, 49 are highly significant, and the top two elements have exceedingly high substitution rates in the human lineage. Some of the properties of the top 49 regions were reported in related work [7], but the evolutionary forces that shaped these regions remain unknown. The goal of this study is to investigate the properties and evolutionary histories of the larger set of 202 HARs in an effort to understand the mechanism(s) by which a highly conserved genomic region can change dramatically in a few million years.

## Results

### Vertebrate Conserved Elements Are Mostly Non-Coding

In order to focus on human-specific changes that have functional importance, we first identified a set of genomic regions which are at least 100 bp in length and identical between chimp *(P. troglodytes),* mouse *(Mus musculus),* and rat *(Rattus norvegicus)* in at least 96% of alignment columns. Evolutionarily conserved regions such as these are expected to be under negative selection to preserve function, because neutrally evolving DNA would have many more nucleotide changes between primates and rodents. Recent findings confirm that highly conserved non-coding elements indeed often play important roles, such as regulating expression of nearby developmental transcription factors [28–31]. Filtering to remove misaligned paralogs, assembly errors, and human pseudogenes produced a set of 34,498 conserved regions. It is important to note that the human genome sequence is *not* used to define the conserved regions, so that the subsequent analysis of human changes is independent of how they were selected.

Bioinformatic analysis of the 34,498 predicted functional elements shows that they are very similar to previously described highly conserved elements in the human genome [32–34]. Only 19.6% overlap coding regions of human genes, while the remaining non-coding regions are mostly intergenic (45.4%) and intronic (31.0%) with a small percentage in UTRs and known non-coding RNAs. Analysis of the Gene Ontology (GO) [35] categories associated with the closest genes to these elements reveals enrichment for transcription factors, DNA-binding proteins, and regulators of nucleic acid metabolism. Development, neurogenesis, and morphogenesis are also significantly overrepresented among these genes. Similar categories are enriched in other studies of highly conserved non-coding elements (e.g., [32]). Interestingly, although only chimp, mouse, and rat were used to define the regions, the majority are present in the draft genomes of more distantly related vertebrates with high (mean = 86.8%) percent identity to human (Table S2). This is particularly striking given that these genomes are incomplete, and the vast evolutionary distance renders all but the most evolutionarily constrained DNA segments unalignable. This level of evolutionary conservation strongly suggests these elements are functional. It also motivates the use of all fully sequenced vertebrate genomes for assessment of acceleration in each region.

### Human Acceleration in Predicted Functional Elements

We ranked the set of 34,498 conserved elements based on evidence of accelerated substitution in the human lineage as quantified by the LRT statistic. The LRT was applied to a multiple alignment of up to 12 vertebrates (17 minus chimp, macaque, mouse, rat, and rabbit) plus the parsimony-inferred chimp-human ancestor, which is used to separate changes that happened before the human-chimp divergence from those that happened after divergence. The two likelihoods in the LRT statistic are each a version of a genome-wide model for conserved sequences, which we call the CONS model, scaled to the region. A large LRT statistic indicates that the multiple alignment for that region is more likely under a model with acceleration in the human lineage than under a model with the same relative rates of substitution as the CONS model. Statistical significance was assessed via simulation from a null model with no acceleration in the human lineage, and empirical *p*-values from these simulations were adjusted for multiple comparisons using the procedure of Benjamini and Hochberg [36] for control of the false discovery rate (FDR). There are 202 regions with genome-wide FDR adjusted *p* < 0.1 (median LRT statistic = 5.06), and 49 of these have FDR adjusted *p* < 0.05 (median LRT statistic = 7.72). We named them HAR1 to HAR202. A table summarizing their properties is available at http://rd.plos.org/pgen_0435_0002.

The 202 HARs resemble the full set of 34,498 conserved regions. They have high levels of conservation across the vertebrates, and they are mostly non-coding (66.3% intergenic and 31.7% intronic, with just 1.5% overlapping coding genes). While none of the HARs overlaps a known non-coding RNA gene, 88 of the 202 elements are predicted to have an RNA secondary structure by the phylogenetic method implemented in the program EvoFold [37], and 12 of these have substitution patterns that provide statistically significant evidence for secondary structure based on random permutation experiments (Table S4). Levels of acceleration are similar between coding and non-coding elements, as well as among all types of non-coding regions. The set of nearest genes to these elements is enriched for the same GO categories as the full set of conserved regions, suggesting possible roles in transcriptional regulation for many of the accelerated elements. These data are consistent with, but of course do not prove, the King and Wilson hypothesis [8] that most significant changes between human and chimp have been regulatory.

In some of the analyses that follow, we focus on HAR1 through HAR5 as a special case, because these five elements all have LRT statistics smaller than any of the datasets that we simulated from the null model (adjusted, *p* < 4.5*e*−4). Some details about these regions are given in Table 1 and Table S5. The biology of these most extremely accelerated elements is currently under experimental investigation and is beyond the scope of this paper. Some bioinformatic clues are noted in the Supporting Information and Figures S3–S7; findings about HAR1 are reported in a companion paper [7]. We also at times focus specifically on HAR1 and HAR2. These two regions are both dramatically changed in human, with estimated substitution rates about twice as high as any other HAR.

**Table 1:**
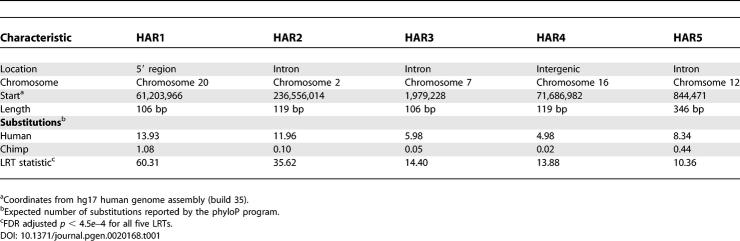
Details of HAR1–HAR5

### From Very Slow to *Very* Fast

In order to investigate substitution rates in individual lineages, we computed the posterior expected number of substitutions on each branch of the 17 species tree using the method described in Siepel et al. [38]. The normalized human substitution rate exceeds the rate in the chimp-rodent phylogeny in all of the HARs, as expected. In HAR1–HAR5, the average estimated human substitution rate per site per million y is 26 times higher than the chimp-mouse rate (Table S5). Directly comparing substitution rates per site in the human and chimp branches (over the same period of evolutionary time), the human rate is an average of seven times higher than the chimp rate in HAR1–HAR5 (Table S5) and exceeds the chimp rate by more than 30% in all but three (1.5%) of the 202 HARs.

The initial analysis of the chimpanzee genome [1] included an evaluation of human-chimpanzee divergence in 10-Mb windows covering the genome. Using the same set of 10-Mb regions, we compared human-chimp divergence in windows that contain one of the HARs, with that in windows which do not contain a HAR. Median divergence is slightly higher in the top 49 HARs (1.33%) than in the remainder of the 202 HARs (1.26%). Windows containing HARs (top 49 and all 202) have significantly higher divergence rates than other windows (median = 1.22%, Wilcoxon rank sum, *p* = 1.2*e* −6). This result suggests that HARs may lie in regions of the genome that are more prone to nucleotide change. Nonetheless, an increase in regional divergence on the order of 0.05% alone cannot explain the extreme levels of acceleration seen in the HARs. Thus, while 64.5% of HARs fall in 10-Mb windows with divergence levels above the median for the whole genome (compared to an expected 50%), the HAR elements themselves are significantly more diverged from chimpanzee than surrounding sequences (HAR element versus rest of 10-Mb window; Wilcoxon rank sum, *p* < 1*e* −15).

Next, we sought to determine how the human substitution rate in HARs compares to neutral expectation. Because the HARs are so conserved in the chimp-rodent phylogeny, it is possible that we might simply have identified relaxation of functional constraint in most cases. To distinguish neutral drift from directional selection, we compared the estimated human substitution rate in each HAR to estimates of the background rate based on 4-fold degenerate sites (4d sites) from ENCODE [39] regions across the genome. Our 4d sites-based estimate of the background substitution rate in the human-chimp tree (average of rates in the human and chimp branches) is approximately 0.0065 substitutions per site genome-wide and 0.009 in last band of chromosome regions. Neutral rates in last bands are identical between human and chimp, whereas the human rate is slightly lower than the chimp rate genome-wide (0.006 versus 0.007). The estimated genome-wide background rate on the human lineage (1.2*e* −3 substitutions per site per million y) agrees well with the rate given in Nachman and Crowell [40].

**Figure 1:**
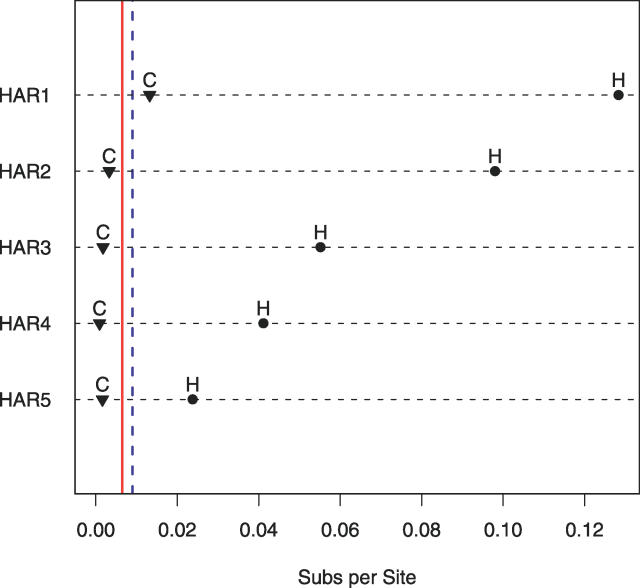
Comparison of Substitution Rates in HAR1–HAR5 For each HAR element, the estimated substitution rate is indicated by a circle for the human lineage and by a triangle for the chimp lineage. As a benchmark, background human-chimp substitution rates estimated from 4d sites in ENCODE regions [39] are marked with vertical lines, solid red for the genome-wide neutral rate, and dotted blue for the neutral rate in final chromosome bands. The chimp rates in all five elements fall well below the human rates, which exceed the background rates by as much as an order of magnitude. H, human; C, chimp.

The human substitution rate exceeds the human-chimp neutral rate in 201 of the 202 HARs, whereas the chimp rate does so in only 33 HARs (16%). This evidence suggests that the HAR elements were not created by relaxation of functional constraint. Among the 12 HARs that fall in the last band of their chromosome arm, all have a human substitution rate that also exceeds the neutral rate in final chromosome bands, whereas only two (17%) have a chimp rate that does so. Thus, this conclusion holds even if we take into account the faster neutral rates near the ends of chromosomes. Figure 1 compares the human and chimp rates in HAR1–HAR5, where the differences are extreme. The human rates in these elements are all exceedingly improbable under a Wright-Fisher independent sites model using either the genome-wide or the chromosome-ends background rate as the null model (Table S6).

In studies of molecular evolution, the index of dispersion (i.e., the ratio of the variance in the number of substitutions on a lineage to the mean number) is used to assess evidence against the neutral model of nucleotide evolution. We computed the index of dispersion for all 202 HARs using the human, chimp, and mouse sequences. In HAR1–HAR5 the index of dispersion is much larger than the expected value of 1 under the neutral model, particularly after adjustment for lineage effects (e.g., generation times, deviations from a star phylogeny) (Table S7). Combined, HAR1–HAR5 have a weighted index of dispersion equal 9.23 based on the method of Gillespie [27] (*p* = 0.018, by simulation). Because selection tends to elevate the index of dispersion of molecular evolution, these data are compatible with strong selection on the human lineage. They could also result from strong BGC.

### Human-Specific Changes Confirmed by Resequencing

We resequenced HAR1–HAR5 in the 24-member subset of the National Human Genome Research Institute (NHGRI) Polymorphism Discovery Resource Panel [41] and in five non-human primates (chimp, orangutan, gorilla, crab-eating macaque, and spider monkey). Table S10 lists which primates were successfully sequenced for each element. Results for HAR1 have been reported in [7].

In no case did the resequenced primate sequences disagree with the chimp assembly at a base where human differed from chimp and rodents (Figure S8). In addition, all individual sequence reads from the National Center for Biotechnology Information trace repository agree between chimpanzee and rhesus macaque *(Macaca mulatta)* at all bases where human differs from chimp and the rodents. These findings confirm that all of the observed human-specific changes in HAR1–HAR5 occurred after human diverged from chimp.

All changes in HAR1–HAR4 appear to be fixed in the human population (Table S11). Seven of the eight human-specific changes in HAR5 are also fixed in the panel. The site hg17.chr12:844,587, however, is polymorphic. Our data suggest that the G in the human genome assembly is the derived allele, which is almost fixed in the human population. An additional human polymorphism was found at hg17.chr12:844,665, where the human assembly has the ancestral T allele. If the more common C allele had been in the human assembly, this would have been counted as an additional human-specific difference (since chimp and all other amniote assemblies are T). Our results are consistent with publicly available polymorphism data. Thus, we conclude that the observed changes in HAR1–HAR5 arose and became fixed (or are becoming fixed in the case of HAR5) in the human population since the human and chimpanzee lineages diverged.

### Evidence of Positive Selection

Human polymorphism data also allow us to test for a recent selective sweep in the regions around the HARs. Selective sweeps result from the “hitch-hiking” effect, which reduces polymorphism (relative to divergence) in regions that have a site under strong directional selection because the haplotype containing the selected site sweeps through the population as the selected allele becomes fixed [42,43]. Using publicly available single nucleotide polymorphism (SNP) data from dbSNP, we performed the HKA test [12] for each of HAR1–HAR5 and found no evidence of departures from neutrality for HAR3, HAR4, and HAR5. HAR1 and HAR2, however, did show significant departures from the neutral model (*p* < 1.0*e* −4 and *p* = 6.0*e* −4, respectively). This suggests that there may have been some positive selection in the regions containing our two most significant elements.

To further investigate directional selection, we applied a novel coalescent-based approach to directly evaluate the probability of a selective sweep in the regions of interest. This approach first uses observed polymorphism and divergence data to estimate the time of species divergence between human and chimp, and then employs this estimate to evaluate the probability of the observed polymorphism, given divergence, in each region. Again, we found no evidence for selection around HAR3–HAR5, but polymorphism around HAR1 and HAR2 was determined to be significantly lower than expected under a range of demographic scenarios and sample sizes, indicating recent selection (Table S8). We repeated this analysis over a range of window sizes (1 kb–10 kb) and found evidence of a sweep around HAR1 and HAR2 at all scales, although the tests only reached statistical significance for windows ≤5 kb. The footprint of a selective sweep depends on the selective coefficient and (inversely) the recombination rate [43]. Hence, the relatively small footprints observed around HAR1 and HAR2 may indicate weak selection (*s **≈*** 1*e^−4^*), although they could also be explained by high recombination rates, as might be expected in regions near the telomeres.

Together, these two different approaches suggest that the regions around HAR1 and HAR2 may have undergone recent selective sweeps in the human lineage. These findings must be interpreted with extreme caution, however, because directed resequencing of a 6.5-kb region around HAR1 found no skew in the folded site frequency spectrum [7] and high levels of polymorphism compared to the genome-wide distribution. In addition, the publicly available dbSNP data employed in the analyses here come from a combination of different studies and undoubtedly suffers from various ascertainment biases [44,45] (Text S1). Thus, whether or not a selective sweep occurred in either HAR1 or HAR2 cannot be decided conclusively from currently available data.

### Striking Substitution Bias from Weak to Strong BP

We observe a dramatic bias for weak to strong (AT to GC, or W→S) nucleotide pair changes in HAR1–HAR2, with 23 out of 24 changes from AT to GC base pairs and none from GC to AT pairs (Table 2). Among HAR3–HAR5, the bias is still present but less extreme (12 out of 19 changes W→S). This bias is seen in both transitions and transversions. In addition, HAR1, HAR3, and HAR5 are all located in the final (distal) band of their chromosome arms (and HAR2 is <550 kb from the final band) so that recombination rates may have been higher in these regions [46,47]. HAR1–HAR3 and HAR5 all lie in larger regions, within which at least 50% of substitutions are W→S in human. These vary in terms of their size (~400–1,200 bp) and bias (Table 2). Based on permutation experiments we found that regions of size ~1 kb around HAR1 and HAR2 are significantly more biased than the surrounding 100 kb. This suggests that the process that generated this bias acted over a region of about 1 kb. The region around HAR4 does not show a W→S substitution bias. Notably, HAR4 is also the only one of HAR1–HAR5 that is not located in the distal end of a chromosome arm.

**Table 2:**
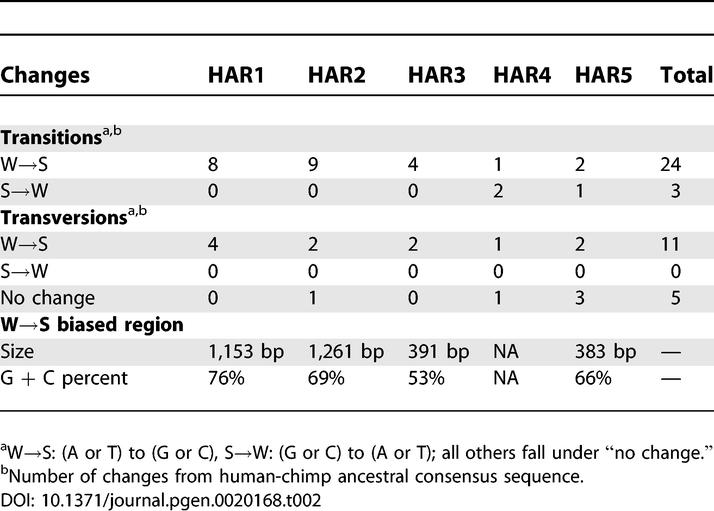
Weak to Strong Bias

**Figure 2:**
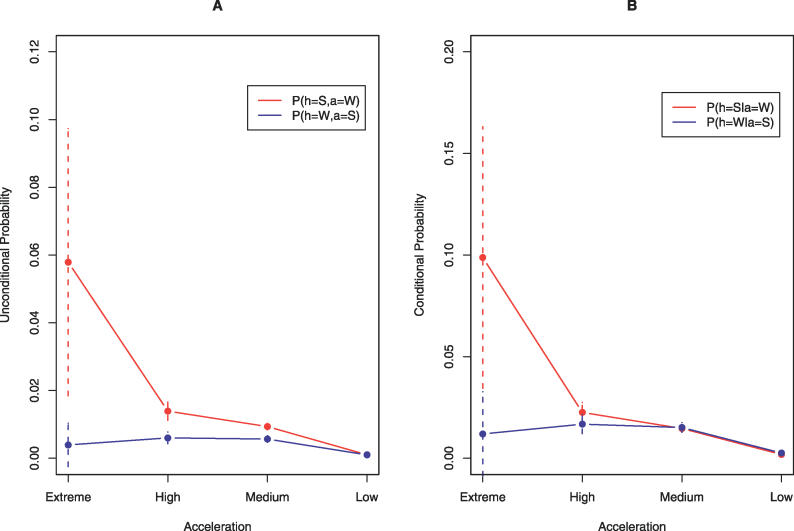
Substitution Bias and Acceleration W→S substitutions (red) increase with acceleration, while S→W substitutions (blue) do not. (A) Proportion of all bases that have W→S and S→W changes versus acceleration in our genome-wide scan of 34,498 elements. The mean proportion of each type of substitution is plotted for four groups based on the amount of acceleration as quantified by the LRT: extreme (*p* < 4.5*e−4)*, high (4.5*e−4 ≤ p* < 0.05), medium (0.05 ≤ *p* < 0.1), and low (*p* ≤ 0.1). These groups correspond to HAR1–HAR5, HAR6–HAR49, HAR50–HAR202, and the remaining ~34,000 conserved elements. The normal 95% confidence interval for each mean is shown with dotted lines. These are estimates of the unconditional probability *P(human = S, ancestor = W)* that a base is strong in human and weak in the ancestral consensus sequence, and vice versa. The differences between substitution types are statistically significant in the extreme and high groups. (B) The same plot, but dividing by the proportion of ancestral bases that are weak or strong. These are estimates of the conditional probability *P(human = S| ancestor = W)* that a base is strong in human, given that the ancestral base is weak, and vice versa. The differences between substitution types are significant in the extreme group only.

In order to investigate W→S bias beyond the five fastest evolving elements, we counted the number of each type of nucleotide change for all 34,498 conserved elements. Grouping together counts for changes that are W→S, S→W, and neither, the proportion of all human substitutions in each category can be computed. W→S substitutions (~50%) outnumber S→W substitutions (~35%) by more than 40% throughout the top 5,000 most accelerated conserved elements, while S→W changes dominate among the remaining less accelerated elements (Figure S2). Among the 202 HARs, W→S substitutions are even more frequent (57% W→S versus 29% S→W). These proportions can be compared to their expected values. Under the CONS model, the genome-wide expected frequency of S→W substitutions is 20% higher than that of W→S substitutions. This is consistent with studies indicating that the substitution bias in the genome as a whole is driving it to become more AT-rich [15–17]. The expected proportion of W→S is just 38%. In fact, there is a clear association between accelerated substitution rate and bias toward W→S substitutions (Figure 2). This is still true (though no longer significantly so) after conditioning on the ancestral base, which accounts for possible effects of compositional bias in the ancestral genome. The bias is particularly extreme in the case of HAR1 and HAR2, though the trend continues through at least the top 49 HARs. Thus, a disproportionate number of the additional human substitutions in the most accelerated HARs are from AT to GC bp, and some (but not all) of this bias is due to variation in the base composition of the ancestral genome in these regions.

We also found that the top 49 HARs are 2.7 times as likely to fall in the final band of their chromosome compared to the remaining ~34,000 conserved elements (Fisher's exact test, *p* = 0.024). The full set of 202 HARs, however, is not disproportionately located in final bands of the chromosomes. Both the observed W→S substitution bias and the tendency to be located in terminal chromosome bands are equally prevalent among the coding and non-coding elements in the accelerated set. Similarly, there is no difference between intergenic and intronic non-coding elements in either trend. This suggests that the effect is regional rather than tied to specific genes.

We performed an additional evaluation of W→S bias genome-wide. Among chimp-human nucleotide differences, only about 15% are AT in chimp and at least one rodent, but GC in human, indicating a very likely W→S substitution on the human lineage. A simple binomial test based on this rate was applied to look for significant W→S bias in approximately 1 million regions in the human genome. We found that regions containing HAR1–HAR3 were among the most significant (ranked 4, 63, and 49, respectively, out of ~1 million regions). HAR1 was, in fact, statistically significantly biased, even after a genome-wide multiple testing correction (FDR adjusted, *p* = 9.8*e* −5). This finding further underscores the strong correlation between rapid and biased regions of change.

Together, these results suggest that BGC may have contributed to the evolution of the HAR regions; particularly the most dramatically accelerated ones [15–17]. An alternative explanation, also compatible with the observed bias, is selection for nucleotide composition to maintain isochores, as originally postulated by Bernardi and colleagues [19,20]. However, the scale of the bias, which appears to be on the order of 1kb, is more consistent with BGC than isochore-related processes.

### HARs Associated with Elevated Recombination Rates

Due to the possible involvement of BGC in the evolution of the HARs, we sought to determine whether HARs are found in regions with higher than expected recombination rates. We examined genome-wide SNP data from Hapmap (Phase I, release 16c.1) [48] and Perlegen [49,50]. We found that 34 (17%) of the 202 HARs and 11 (22.4%) of the top 49 HARs fall in predicted Hapmap or Perlegen recombination hotspots identified by the package LDHot [51] (SNP Recomb Hots track on UCSC browser). This is slightly more overlap than expected by chance alone (*p* = 0.152 for the 202 HARs and *p* = 0.072 for the top 49, based on randomly placing HARs on the chromosomes 1,000 times). Next, we examined fine-scale recombination rates estimated with the package LDHat [51]. The mean recombination rate in HARs is 34% higher than the genome-wide average in the Perlegen data (Wilcoxon, *p* = 0.02), but it is not higher than the genome-wide average in the Hapmap data. The mean recombination rate in the top 49 HARs is higher than the full set of 202 HARs, 4.4% above the genome-wide average in the Hapmap data, and 68.0% higher than the average in the Perlegen data, though the differences are not statistically significant. Thus, genomic regions containing HARs have an increased probability of having elevated recombination rates in the current human population, although the effect is not dramatic.

## Discussion

We have scanned the whole human genome and identified the most extreme examples of recent, rapid molecular evolution. After careful screening to remove alignment and assembly errors, we found 202 significantly accelerated elements. In this work, we have extensively characterized the bioinformatic properties of the HARs, paying particular attention to the most extremely accelerated elements. The ranked list of HARs is a rich source of genomic regions for further study and functional characterization. Some of this work has been undertaken for HAR1 [7], showing that it is a small structural RNA expressed during development of the neocortex.

The 202 HARs resemble the full set of conserved regions. The majority are located in conserved non-coding regions. Many are found in the introns of, and adjacent to, genes annotated with GO terms related to transcription and DNA binding. These findings are in agreement with the hypothesis, first proposed by King and Wilson in 1975, that the majority of chimp-human phenotypic differences can be explained by differential control of transcriptional networks [8,52], which may be expected to occur primarily in the non-coding DNA.

Changes in the human lineage could represent either the loss of a functional element [53] or a change in its function. By comparing estimates of the human substitution rate (genome-wide and in the final bands of chromosomes), we found that all of the HARs have been evolving at faster than neutral rates. The two most accelerated regions, named HAR1 and HAR2, have exceedingly high substitution rates in the human lineage, implying an approximately 4-fold increase in selective coefficient if positive selection were the only explanation for the acceleration (Text S1). However, detailed examination of these data indicates that forces other than selection for random mutations that increase fitness in specific functional elements may be at play in the most rapidly evolving regions. Careful analysis is needed to tease apart these disparate forces. We observed a strong correlation between acceleration and bias toward AT→GC nucleotide pair changes in regions of size from 100–1,000 bp. This bias occurs equally in intronic, intergenic, and coding elements. Acceleration and bias are more frequent in regions in the final band of their chromosome arm. Interestingly, the orthologous regions of HAR1 and HAR5 are also in final bands in other mammals. Since the sequence of these elements is highly conserved across the vertebrates, they appear to have been very stable for an extended evolutionary period despite their location near chromosome ends, before being radically reworked during the last ~5 million y of human evolution. The general association between increased divergence rates and location near chromosome ends is consistent with a recent whole-genome comparison of chimp and human [1] that found increased divergence (15% greater than the rest of the chromosome on average) in the terminal 10 Mb of each chromosome. Our results go further, indicating that regions at the ends of chromosome arms are not uniformly or constantly changing more rapidly than other regions, but rather, acceleration can be a sudden, extreme and uneven process, with clusters of rapid, biased changes occurring in local W→S regions of ~1 kb, even in elements that are otherwise usually highly resistant to change.

BGC is one possible factor in this process. There is more recombination at the distal ends of chromosome arms, and the location of recombination hotspots is known to change rapidly during evolution. In particular, it differs widely between human and chimp [48,54]. Hence, we do not necessarily expect there to be an association between HARs and current recombination rates. Nonetheless, we do find more HARs than expected based on genome-wide data in regions with elevated recombination rates. Recombination can also be mutagenetic [18,46,55]. Recombination hotspots appearing some time in the last 5–6 million y could thus provide a mechanism for both the biased fixation of G and C nucleotides in the pre-human population and the polymorphic sites needed to start this process. In particular, the error prone repair of recombination-associated double-stranded breaks in the DNA could produce clusters of mutations over a relatively short period of evolutionary time, either together during a single recombination event or as independent mutations. BGC could then drive the rapid fixation of the derived GC alleles in the population. Note also that there is a marked increase in the number of segmental duplications and rearrangements created by non-homologous end-joining and interlocus gene conversion in human subtelomeric regions [56]. This also implies an increased number of double-stranded breaks, which in combination with BGC could have contributed to the effects we see. A similar hypothesis was recently put forth by Spencer et al. [57] to explain a fine scale (2–4 kb) association between recombination and diversity observed on human Chromosome 20.

Increased positive selection in these regions is an alternative explanation; if rather than (or in addition to) selection for random fitness-increasing changes in specific functional elements, there is selection for increased G + C content in larger isochores, as proposed by Bernardi and colleagues [19,20]. In this theory, neutral and weakly deleterious changes drive a large region (>100 kb) to a critical point, below which the G + C content cannot fall without significantly deleterious effect. At that point, W→S substitutions in the region suddenly gain a selective advantage, and may sweep through the population. The effects of the sweep on polymorphism and divergence would be similar to those that result from selection for specific, non-isochore-related advantageous alleles in genes. With the data at hand it would be difficult to distinguish this from selection for specific changes in functional elements. However, we may still hope to distinguish selection in general from BGC.

BGC mimics selection in many ways [58], so that most tests cannot distinguish them. However, the size of a gene conversion event (i.e., track length during DNA repair) is thought to be geometrically distributed with a mean of several hundred bp in humans [59], whereas the domain of selection in a sweep can be tremendous [43]. Under the selected-isochore model, selective constraints are shared over larger regions (hundreds of kb). Thus, we do expect quite a different sweep signature for selection versus BGC. The regions around HAR1 and HAR2 that have significantly reduced polymorphism relative to divergence are ~5 kb, which is more consistent with selection than with BGC. This does not rule out the possibility that large transient mutational hotspots created short-lived increases in mutation rate in these regions, increasing divergence without affecting current levels of polymorphism and thereby simulating a selective sweep [18], or that there was an unusually extended BGC event. However, it does at least suggest that selective forces were at work in driving the changes in these regions; albeit, not on the scale of hundreds of kb. In cases like HAR1, where the DNA that exhibits the W→S substitution bias is transcribed, another possibility is selection for increased gene expression [21].

Although we found a reduced ratio of polymorphism to divergence suggestive of positive selection around HAR1 and HAR2, directed resequencing of 6.5 kb around HAR1 produced a folded-site frequency spectrum that is consistent with the neutral model [7] and does not suggest a recent selective sweep. It is important to note that these two analyses of selection at HAR1 use different data (1 Mb of publicly available SNPs here versus 6.5 kb of resequencing in single populations in [7]) and hence different methods. The HKA and coalescent-based tests that we performed with publicly available SNPs were not feasible with the resequencing data which lack a suitable control region sequenced in the same populations. Hence, allele frequencies in the observed resequencing data were compared to theoretical expectations under the neutral model. In contrast, we perform a more nonparametric, empirical analysis here, in which each focal locus (centered on a HAR element) is compared directly to the surrounding genomic environment. In addition, the use of divergence data as a benchmark for levels of polymorphism may improve our ability to detect a sweep when both diversity and skew in allele frequencies have mostly recovered (after ~ *N_e_* generations, where *N_e_* is the effective population size). One interpretation of these results is that selection most likely occurred, but that it appears to have acted long enough ago (>250,000 y) or been weak enough (as suggested by the ~5-kb footprint) that it could not be detected in the site-frequency spectrum observed in the resequencing data analysis. The presence of compensatory substitutions in the RNA structure of HAR1 [7] supports this hypothesis. Unfortunately, however, our ability to confidently reject the neutral model in the HARs is reduced by the likely presence of ascertainment biases present in the publicly available data used here.

Thus, while we can pinpoint the locations of the most rapidly accelerated elements in the human genome, we cannot determine the exact cause of this acceleration with present data. Since we searched the entire genome for the most extreme cases, there is the distinct possibility that changes in the regions we observe result from a combination of multiple evolutionary processes, perhaps including BGC and a selection-based process. In particular, the intensity of the increased selective coefficient in the most dramatically accelerated elements supports the hypothesis that multiple evolutionary forces have contributed to these fastest evolving elements in the human genome.

## Materials and Methods

In order to be concrete, we describe our methods in terms of identifying human-specific changes in genomic regions conserved between chimp, mouse, and rat. Note, however, that the approach is general and could also be used, for example, to identify rat-specific changes using mouse, human, and dog or various other combinations.

### Sequence data.

We use the following publicly available genome assemblies: Homo sapiens (NCBI build 35, May, 2004, hg17), Pan troglodytes (NCBI build 1, November, 2003, panTro1), Macaca mulatta (January, 2006), Mus musculus (NCBI build 35, August, 2005, mm7), Rattus norvegicus (Baylor HGSC version 3.1, June, 2003, rn3), Oryctolagus cuniculus (May, 2005 draft), *Canis familiaris* (May, 2005), *Bos Taurus* (March, 2005), Dasypus novemcinctus (May, 2005 draft), Loxodonta africana (May, 2005 draft), Echinops telfairi (July, 2005 draft), *Monodelphis domestica* (June, 2005), Gallus gallus (February, 2004), Xenopus tropicalis (October, 2004), Tetraodon nigroviridis (February, 2004), Danio rerio (May, 2005), and Takifugu rubripes (August, 2002). Analysis of vertebrate conservation uses *Canis, Gallus, Xenopus,* and *Tetraodon*. All of these genome sequences and the annotations (e.g., genes, mRNAs, ESTs, SNPs) used to interpret our results are available on the UCSC Genome Browser (http://www.genome.ucsc.edu). In addition, we use individual reads from the NCBI trace repository to check the genome assemblies in crucial regions and to provide data from additional species regarding how far back in evolutionary time individual conserved elements may have been under negative selection.

We first apply the MULTIZ alignment program to the whole genome sequences of up to 17 vertebrates with human as the reference sequence [60].We omit from further analysis all alignment columns containing gaps. Bases in CpG dinucleotides are also excluded, since these are known to have a particularly high mutation rate (from the methylated form of CG to TG and CA) [61] and could therefore violate our assumption of independence between sites and easily skew our results.

We use percent identity to define blocks of consecutive bases for which most nucleotides are perfectly conserved between chimp and the rodents. Table S1 shows the number of genomic regions we identify by varying the minimum block length (50,100,200 bp) and minimum percent identity (90%–100%). Qualitative results of our genome-wide scan, including our most significant findings, do not change substantially between the different sets. We report here on the results for the set of blocks at least 100-bp long and at least 96% identical. There are 34,498 such blocks after filtering. The median length of these blocks in human is 140 bp with range 97 bp (<100 bp due to human deletions) to 1,240 bp. Table S3 shows the distribution of the number of human-specific changes in these blocks. We describe an alternative method for identifying conserved blocks, based on the phastCons program [33] in the Supporting Information. Details of the phastCons elements containing HAR1–HAR5 are shown in Table S9.

We obtain an estimate of the genome sequence of the most recent common ancestor of human and chimp using MULTIZ multiple alignments of human, chimp, and rhesus macaque. In this case (two species with one out-group), the ancestral consensus sequence provides a parsimonious estimate. That is, if the human and chimp bases are identical the ancestor is assigned this base. Otherwise, the ancestor is assigned whichever of the two bases agrees with macaque (if either do agree) and “N” (unknown) if none of the three bases agree. Elements without both chimp and macaque sequence (629 of 34,498) are omitted from further analysis.

### Detecting acceleration in human.

The methods we use to study accelerated substitution rates employ a general time-reversible (REV) single-nucleotide model for molecular evolution [62]. Parameters are estimated from an independent dataset of conserved alignments by maximum likelihood (ML) using the phast library, available from acs4@cornell.edu. Details are given in Figure S1. We call this fitted model the CONS model.

We rank genomic regions based on evidence for accelerated substitution rate in human using an LRT. The LRT statistic for acceleration in human compares the likelihood of the alignment data under two models. In the first model, the human substitution rate is held in proportion to the other substitution rates in the tree. In the second model, there is an additional parameter for the human substitution rate which is allowed to be relatively larger than the rates in the rest of the tree. Both models are fit to each alignment by scaling the CONS model. In the first case, scaling involves increasing or decreasing the substitution rates throughout the whole tree in equal proportions. In the second case, the subtree containing the human branch is scaled separately from the supertree containing the rest of the branches. Then, the LRT statistic is the log ratio of the likelihood of the observed data under the second model (with human rate parameter) to the likelihood under the first model (with proportional rates). Large values of the LRT indicate more evidence for acceleration in the human lineage. Because the distribution of the LRT statistics is unknown in this setting, we assess their significance by simulation from the equal rates model. We generate 1 million simulated null alignments of variable lengths (median = 140, as in the observed data). For each of the 34,498 regions, an empirical *p*-value is given by the proportion of simulated datasets with a larger LRT statistic. For the LRT analysis, we omit chimp, macaque, mouse, rat, and rabbit from the multiple alignments (plus the branch between the chimp-human ancestor and the next closest species) so that the test is independent of the method used to select conserved regions. The chimp, mouse, and rat genomes were used explicitly in the identification of conserved regions, while the rabbit and macaque genomes lie alone at the ends of branches that were also used. We note that the macaque and rabbit leaves are effectively independent from the rest of the tree (after chimp, mouse, and rat have been omitted) and that their elimination had almost no effect on the results.

For comparison of substitution patterns on different branches of the primate-rodent tree, we estimate the expected number of substitutions as follows. At a given locus, we compute the posterior expected number of substitutions on each branch of the 17 species tree using the CONS model as prior distribution. We use the program phyloP with option –subtree (phast library) to compute the posterior estimates [38]. This method accounts for all possible labelings of the ancestral nodes and for multiple substitutions per branch. The (rounded) posterior mean gives a conservative estimate of the number of substitutions, because the posterior distribution will be biased towards the prior, which is a model with low substitution rates here. The number of substitutions can be scaled by the element length and the evolutionary time represented by the branch, giving substitutions per bp or per bp per million y. We use the following branch lengths in millions of y (my): human = chimp = 5 my, branch from the chimp-human ancestor to the rat-mouse ancestor = 127 my (based on a human-mouse ancestor 75-my ago), and rat = mouse = 18 my [63,64].

### Filtering and validation.

Before performing the statistical analysis, we apply the following filters to the set of conserved regions. First, we use the human-mouse chains [65] and other information from the UCSC browser database [66] to exclude any regions that (i) are not contained in a mouse synteny “net” or (ii) are not contained in a chimp alignment “chain.” The requirements of mouse “synteny” and continuous chimp alignment provide general assurance that we are looking at orthologous bases rather than alignment errors. Second, we remove regions that lie in a known human pseudogene. Human psuedogenes are a particular instance where the human sequence will align to the sequence from the original gene in chimp and other species. Because the human sequence is not under the same functional constraint as the original gene, there will likely be an increased rate of substitution in the human sequence as it evolves neutrally. In this analysis, we are not interested in this process and chose to focus on human-specific changes in uniquely orthologous sequence only. Finally, we look for examples of human paralogs. Paralogous sequences, like human pseudogenes, can cause errors in the multiple alignment if the human copies are not matched correctly with the copies in the other species or if there is only one copy in the other species. We remove any element that overlaps a chain in the “self chain” track (BLASTZ of the human genome against itself) available at http://www.genome.ucsc.edu.

After identifying the most significantly accelerated elements, we perform additional manual screening of the top hits. This includes performing a BLAT search of the DNA for the element against the whole human genome to double check for paralogous sequences. We also examine the multiple alignments for examples where the human-specific substitutions are within three alignment columns. It is possible that these changes occurred together and represent only one evolutionary event. When these are found, we conservatively repeat the statistical analysis removing all but one of the putatively dependent bases, checking if the results depend on which base is retained. This simulates there having been only a single event at each site. Finally, we use the NCBI BLAST server (http://www.ncbi.nlm.nih.gov/BLAST) to compare the human nucleotide sequence of each of our most significant elements to all of the reads in the NCBI trace repository from any species with substantial coverage. This allows us to confirm that the sequence from the species in our multiple alignments is indeed the best match from all of the reads of their genomes and thereby discover any assembly errors.

In addition to filtering based on bioinformatic data, we also remove from our analysis any elements where human resequencing suggests an error or rare variant in the human genome reference sequence or where primate resequencing suggests an ambiguous evolutionary history for the region.

### GO categories.

For each accelerated element, the nearest gene in either direction is identified without any filter on distance between the element and the gene. Restricting the analysis to genes within 1 Mb of the element does not subjectively change the findings. The GO [35] categories associated with each gene are obtained from the GO website: http://www.geneontology.org. For each term present in the list of genes nearby conserved elements, a hypergeometric test is used to compare the proportion of genes in the conserved list annotated with that term to the proportion genome-wide.

### Resequencing.

Primers are designed to amplify the genomic region surrounding each of the fastest evolving elements (Table S10). For HAR2–HAR5, a single set of primers is expected to work on chimpanzee and macaque DNA as well. In the case of HAR1, specific primers are designed for human, chimpanzee, and macaque. These primers are used for PCR using genomic DNA from the 24-member subset of the NHGRI Polymorphism Discovery Resource Panel [41] as a template. PCR is performed using 4 ng/μl of the appropriate genomic DNA and UltraPfu polymerase (Stratagene, La Jolla, California, United States) under conditions recommended by the manufacturer. In addition, the primers are tested on a panel of primate DNA including chimpanzee, crab-eating macaque, gorilla, orangutan, spider monkey, and ring-tailed lemur (six-member primate panel, Coriell Cell Repositories, Camden, New Jersey, United States). PCR products are subjected to sequence analysis using ABI Prism 3730xl sequencers (Seqwright, Houston, Texas, United States). Sequencing results are analyzed using ContigExpress (VectorNTI Suite, Invitrogen, Carlsbad, California, United States). Non-human primate sequences help decide the location of substitutions at bases where human and chimp differ, because if they agree with the chimp genome we are confident that there was a single substitution on the human branch.

### Population genetics.

For each locus, we extract the number of SNPs (segregating sites) and the number of sites where human and chimp have different nucleotides (divergence) from the UCSC Table Browser at http://www.genome.ucsc.edu. Because this dataset pools SNPs from many sources (the various studies available from dbSNP, Perlegen Sciences, and Affymetrix), there may be serious ascertainment problems with this data [44,45]. To attempt to control for variation in ascertainment across SNP data, we performed all of our population genetic analyses at three different assumed sample sizes to explore how sensitive our conclusions might be to ascertainment bias. Qualitatively, our conclusions were robust to assumptions of sample size; however, interpretation of our results using the publicly available SNP data deserves caution. The significance values we report in the main text are based on a sample size of 50, which we conservatively estimate to be approximately correct for most SNPs.

We examine linkage disequilibrium (LD) around HAR1–HAR5 using pair-wise measures of *R* ^2^ for all Hapmap SNPs in a 1-Mb window centered at the element. HAR1 is in a particularly strong LD block about 10-kb wide. The other HARs are also near SNPs in LD. For all five regions, *R* ^2^ falls below 0.1 for most pairs of SNPs that are 7 kb or more away. The decay in LD is fastest in the HAR1 region.

For each HAR element we perform the HKA test [12] comparing the element plus 1 kb of flanking sequence to the surrounding 1-Mb window of sequence. Two different HKA methods are used. First, we perform the HKA test in direct mode with the DnaSP (v4.0) software. This test employs the chi-squared distribution to assess significance. Second, we assess significance of the same data using an alternative, coalescent-based simulation approach implemented by J. Hey http://rd.plos.org/pgen_0435_0001. This method avoids assumptions needed for the chi-squared distribution to be appropriate. Here, results from the two methods are nearly identical. We report the Hey HKA *p*-values.

Using the same polymorphism and divergence data as the HKA tests, we perform a direct computation of the probability of a selective sweep in the genomic regions containing each of our top four elements. In essence, this is just a coalescent extension of the HKA test that evaluates the probability of a locus having the observed number, or fewer, segregating sites conditional on a ML estimate of the species divergence time and the number of fixed differences at that locus. As human demographic history is known to be complex, we performed this test under standard neutral model, as well as two models of population expansion and one model of a population bottleneck followed by a subsequent expansion. Details are given in the Supporting Information. We repeat the analysis with a range of window sizes from 1 kb to 10 kb. Results from all scales are reported. Results based on 1- to 5-kb windows produce similar results, whereas evidence of selection in HAR1 and HAR2 is less clear at the 10-kb scale.

Data from human, chimp, and mouse are used in the computation of the index of dispersion of molecular evolution. Index of dispersion is computed as the variance in the number of substitutions divided by the mean number across several lineages within a single locus. For each of the HAR elements, we use the ML estimated lineage-specific number of substitutions to compute both the unadjusted index of dispersion and the weighted version based on the method of Gillespie [27]. Weights are used so that all lineages have the same expected number of substitutions, correcting for lineage effects. Rather than estimating these weights directly from our small dataset of five loci, we use the genome-wide estimates of lineage-specific rates from the fitted CONS model (Figure S1). Significance of observed index of dispersion values is assessed by simulation (with 10,000 iterations) of a tree with equal rates on human, chimp, and mouse branches. The rate is the observed mean rate for each element.

### Weak to strong bias.

For each conserved region, the multiple sequence alignment of human, chimp, rat, and mouse is scanned for all sites where human has a different base than chimp, which is identical to both rodents. These sites are very likely cases of derived changes in human. Identified changes are grouped into the following categories: weak to strong, strong to weak, or neither. For each region, two types of probabilities are estimated. Unconditional (or joint) probabilities, e.g., *P(human = S, ancestor = W),* are estimated by the proportion of bases in each category. Conditional probabilities, e.g., *P(human = S, |ancestor = W) = P(human = S, ancestor = W)/P(ancestor = W),* are estimated by the unconditional estimate divided by the proportion of the consensus ancestral bases in the given category (W or S). Next, regions are ordered by LRT statistics and grouped as extreme (*p* < 4.5*e* −4), high (4.5*e* −4 ≤ *p* < 0.05), medium (0.05 ≤ *p* < 0.1), and low (*p* ≥ 0.1) acceleration. Average bias is then compared between groups. As an alternative view of the data, these averages are also computed for sliding windows of ordered regions using a loess smoothing function (based on polynomials of degree 2 with smoothing parameter = 0.75).

In order to study bias genome-wide, we identify a genome-wide set of all blocks of sequence (2,285,015 total) with at least four chimp-human nucleotide differences occurring in a frequency of not less than one per 32 bp and with gaps of no more than 96 bp between differences. An analysis based on higher density blocks produces qualitatively similar results. Multiple alignment data for mouse and/or rat are used to determine the most likely lineage for each difference (where available), and blocks with no chimp-human difference where lineage could be determined are dropped. The remaining blocks (over 1 million) are analyzed for the significance of weak to strong (W→S) substitutions on the human lineage. For each block, a binomial *p*-value is computed from the observed number of substitutions and the number of these that are W→S, using the estimated overall proportion of W→S genome-wide (0.1518).

To examine bias around the most accelerated elements, multiple sequence alignments of human, chimp, and macaque *( M. mulatta)* in the regions around HAR1–HAR5 are generated and scanned for examples of chimp-human differences where macaque sequence agrees with chimp, suggesting a substitution on the human lineage. Macaque is used as the out-group here because it is evolutionarily closer than the rodents, making double substitutions less likely. The identified human changes are typed as weak to strong, strong to weak, or neither. A window of GC bias around each HAR element is then created by expanding the set of human derived changes until another change cannot be added (up or downstream) without creating a run of ten changes with less than 5/10 being G or C in human and A or T in the other primates. The size and percent W→S of this window is computed for each HAR element.

In order to assess the significance of the observed W→S bias around HAR1–HAR5 given local sequence features, we perform simulation experiments in which the order of the observed human-chimp differences in a 100-kb region centered at each HAR are permuted and the bias in windows of ten differences is recomputed across the permuted data. We compare the bias from the observed data to the maximum and average bias for each permuted dataset.

## Supporting Information

Figure S1 CONS Model Estimated REV model fit on the conserved (phastCons) regions of a random 50 Mb of the 17 species whole-genome MULTIZ alignment as described in [7,33]. Branch lengths are in substitutions per base. An unrooted tree was estimated, but it has been rooted for display purposes. G + C percentage is 40.7%. The ratio of the number of transitions to the number of transversions is 1.71. We call this estimated tree (minus the mouse, rat, rabbit, macaque, and chimp branches) the CONS model.
(20 KB JPG)

Figure S2 W→S Bias versus Acceleration The horizontal axis shows rank based on LRT *p*-values, with low-ranking elements being the most accelerated in human. The vertical axis is the average proportion of substitutions. The plotted loess curves (based on polynomials of degree 2, with smoothing parameter = 0.75) represent the smoothed mean over sliding windows across the ranking. The most accelerated elements are largely composed of W→S substitutions (red), and the proportion W→S decreases with decreasing acceleration until S→W substitutions (blue) become more common than W→S substitutions around element 5,000. As expected, there is an inverse relationship between the proportion W→S and the proportion S→W. Substitutions that are neither W→S nor S→W are plotted in green. The expected proportion of each type of substitution is 1/3 under a Jukes-Cantor model. Under the CONS model, the expected proportions are 38% W→S, 47% S→W, and 15% neither. The top 10,000 elements are plotted. Proportions remain essentially constant at the levels at element 10,000 for the remaining ~25,000 elements.
(258 KB PDF)

Figure S3 HAR1 UCSC genome browser shots, human build 35 (hg17). Upper panel shows the *HAR1F-* and *HAR1R*-predicted genes. The human hippocampus-expressed mRNAs and testis-expressed EST are shown. The predicted EvoFold [37] RNA structure overlaps the conserved region. Lower panel shows HAR1 element with transcripts and RNA structure prediction. Conservation across the amniotes is very high.
(4.9 MB PDF)

Figure S4 HAR2 UCSC genome browser shots, human build 35 (hg17). Upper panel shows the entire *CENTG2* gene, plus nearby gene *GBX2.* Lower panel: HAR2 element. Conservation across the vertebrates is very high.
(4.3 MB PDF)

Figure S5 HAR3 UCSC genome browser shots, human build 35 (hg17). Upper panel: the entire *MAD1L1* gene, plus the nearby genes *FTSJ2* and *NUDT1* and several hypothetical proteins. Lower panel: HAR3 element. Conservation across the amniotes is high.
(4.4 MB PDF)

Figure S6 HAR4 UCSC genome browser shots, human build 35 (hg17). Upper panel: the entire *ATBF1* gene. Lower panel: HAR4 element. Conservation across the amniotes is high. A small, predicted RNA structure is shown in the EvoFold track.
(4.1 MB PDF)

Figure S7 HAR5 UCSC genome browser shots, human build 35 (hg17). Upper panel: the entire *PRKWNK1* gene, containing the single exon gene *HSN2.* A hypothetical protein and alternatively spliced isoform are also shown. Lower panel: HAR5 element. Conservation across the vertebrates is high. The three publicly known human SNPs are marked with their rs numbers.
(5.2 MB PDF)

Figure S8 Human and Primate Resequencing Data This shows results of resequencing of the 24-member subset of the NHGRI Polymorphism Discovery Resource Panel [41] and Coriell Primate Panel. Bases in lower case are identical in all sequences. Human diffs (low and high quality) are bold. Bases polymorphic in the human panel are underlined and the more common nucleotide is shown. Assembly, human genome assembly (hg17); human, PDR panel; chimp, chimpanzee; orang, orangutan; spider, spider monkey.
(22 KB PDF)

Table S1 Percent Identity in Chimp-Rodent Alignments Shows counts of the number of blocks in multiple alignments of the chimpanzee, mouse, and rat genomes that are a given length and whose nucleotide sequences are a given percent identical (across all three species). Only nucleotides (A,C,T, and G), and not indels or missing data, count as identical. The number in parentheses gives the count of these blocks that contain at least one alignment column where chimpanzee, mouse, and rat have an identical nucleotide and human has a different one (human diff). The 466 blocks ≥200 bp and 100% identical are analogous to (but not identical to) the ultraconserved elements described in [32], which used the human, mouse, and rat genomes.
(104 KB PDF)

Table S2 Conservation in Additional Species Conservation patterns of 34,497 primate-rodent conserved regions among more distantly related vertebrates. Mean percent identical gives the average proportion of bases that match human (hg17, build 35) among the regions that are present in that species. Average percent identity with human is 86.8%, which is very high compared to typical orthologously alignable DNA (e.g., 67% in mouse [34] and 60% in fish [32]).
(13 KB PDF)

Table S3 Distribution of Human-Specific Changes in Conserved Blocks This shows counts of the number of conserved blocks (out of 34,498) with more than 0–5 human diffs. Human diffs are sites where the chimp, mouse, and rat bases are identical, but the human base is different.
(12 KB PDF)

Table S4 HARs with Significant RNA Secondary Structure Observed score (S) is a linear combination of counts of different types of substitutions. *P*-values are computed empirically by shuffling the columns of the multiple alignment as described in Pedersen et al. [37] (not adjusted for multiple comparisons).
(17 KB PDF)

Table S5 Comparison of Estimated Substitution Rates in HAR1–HAR5 Substitution rates are posterior expected value of the number of substitutions using the method described in Siepel et al. [38] with the CONS model as prior. The human:chimp ratio is the estimated number of substitutions per site in human compared to chimp. The human:(chimp-mouse) ratio is the estimated number of substitutions per site per million y in human compared to the chimp-mouse phylogeny. Note that because rodent generation times are much shorter than human, we expect the rates in y to be smaller in human than in the chimp-mouse if the regions are evolving at the same rate per generation. Branch lengths for scaling rates in millions of y: human = 5 my, chimp-mouse = 150 my. Totals computed by concatenating the five elements.
(14 KB PDF)

Table S6 Significance of Human Acceleration Wright-Fisher independent sites model *p*-values for human substitutions compared to the genome-wide human-chimp background rate based on ENCODE region [39] 4d sites and the human background rate in 4d sites of ENCODE regions in final chromosome bands. Unadjusted *p*-values are reported.
(22 KB PDF)

Table S7 Index of Dispersion in HAR1–HAR5 Weighted index of dispersion computed using the method of Gillespie [27]. *P*-values computed by simulation (not adjusted for multiple comparisons).
(15 KB PDF)

Table S8 Selective Sweep Test *P*-Values Significance of the observed pattern of polymorphism and divergence for each HAR compared to the surrounding 1 Mb. Coalescent-based simulations were performed with four demographic models: the standard neutral model, a model with a recent population expansion, a model with an ancient population expansion, and a model with a population bottleneck followed by expansion. For each HAR, a separate analysis was conducted under each model at each combination of three scales (windows of size 1 kb, 5 kb, and 10 kb) and three sample sizes (*n* = 10, 50, 100). The *p*-values are the proportion of the 10^5^ simulated datasets that have the observed number or fewer segregating sites, conditional on the observed human-chimp divergence and the speciation time estimated from the surrounding 1-Mb regions. Unadjusted *p*-values are reported.
(16 KB PDF)

Table S9 Details of phastCons Elements Containing HAR1–HAR5 Coordinates are from hg17 human genome assembly (build 35). Human differences give the number of bases that do not match the human-chimp-macaque consensus ancestral sequence. The human:chimp ratio is the estimated substitutions rate per million y in human compared to chimp. Rates are estimated using the method described in Siepel et al. [38] with the CONS model as prior.
(25 KB PDF)

Table S10 Resequencing Primers Details of the primers used in this study. H, human; C, chimpanzee, M, macaque; G, gorilla; O, orangutan; S, spider monkey; L, lemur.
(13 KB PDF)

Table S11 Polymorphism Discovery Resource Panel Sequencing Results Resequencing of the 24-member subset of the NHGRI Polymorphism Discovery Resource Panel [41]. Nearly all human-specific substitutions are fixed in the panel, including all low-quality human diffs.
(12 KB PDF)

Text S1 Supplementary Methods and Results (126 KB PDF)

### Accession Numbers

The Swiss-Prot (http://www.ebi.ac.uk/swissprot) accession number for the gene *PDYN* is PO1213.

## Abbreviations

AT: adenine and thymine
BGC: biased gene conversion
bp: base pair
FDR: false discovery rate
4d: 4-fold degenerate
GC: guanine and cytosine
GO: Gene Ontology
HAR: human accelerated region
HKA: Hudson-Kreitman-Agude test
kb: kilobase
LRT: likelihood ratio test
SNP: single nucleotide polymorphism
