# Supplementary material for: Forces Shaping the Fastest Evolving Regions in the Human Genome
Source: PLoS Genet. 2006 Oct 13;2(10):e168. doi: 10.1371/journal.pgen.0020168 (PMC1599772; doi:10.1371/journal.pgen.0020168)
Supplement: Supplementary file 1 [file pgen.0020168.sd001.pdf]

Forces Shaping the Fastest Evolving Regions in the Human Genome

Katherine S. Pollard[1,†,*] Sofie R. Salama[1,2] Bryan King[1,2]
Andrew Kern[1] Tim Dreszer[1] Sol Katzman[1,2] Adam Siepel[1,‡]
Jakob Pedersen[1] Gill Bejerano[1] Robert Baertsch[1]
Kate R. Rosenbloom[1] Jim Kent[1] David Haussler[1,2]

Supporting Text S1

1 Dept. of Biomolecular Engineering and
2 Howard Hughes Medical Institute, University of California, Santa Cruz, CA 95064
† Present Address: Genome Center & Dept. of Statistics, University of California, Davis, CA 95616
‡ Present Address: Dept. of Biological Statistics & Computational Biology, Cornell University, Ithaca, NY 14853
* Corresponding author

S1.1 Human diffs.

We define a *human diff* as a base where (i) chimp, mouse, and rat have the same nucleotide, (ii) human has a different nucleotide, (iii) the ancestral consensus sequence is not in a CpG dinucleotide, and (iv) the chimp base is high quality (Phred score ≥ 30 and in an 11 base window with no indels, no more than 2 human/chimp differences, and all Phred scores ≥ 25). If the chimp base is not high quality, we refer to the base as a *low quality human diff*. The number of human diffs in an element can be compared to the expected number if the element were evolving neutrally in the human lineage: 0.67 diffs per 100bp, based on a genome-wide estimate of P(*human≠chimp* | *chimp=mouse=rat)*. Table S3 contains the distribution of the number of human diffs in our set of 96% conserved regions. Nearly 24% of the regions have at least one human diff. In addition, they contain between 0 and 6 human indel events (mean=0.38), affecting between 0 and 28 bases (mean=0.52).

S1.2 Model for nucleotide evolution.

The methods we use to detect substitution rate acceleration all make use of a fitted molecular evolutionary model. For this purpose, we use a general reversible single-nucleotide model (REV)[1] with parameters estimated from a genome-wide data set of evolutionarily conserved bases constituting approximately 3% of the human genome. These sites were identified in an independent analysis of a 17 species multiple alignment of human, chimp, macaque, mouse, rat, rabbit, cow, dog, armadillo, elephant, tenrec, opossum, chicken, frog, fugu, tetraodon, and zebrafish using the methods described in Siepel *et al.*[2]. Using the 17 species alignments and a topology for their phylogenetic tree, we globally estimate the free model parameters (rate matrix, branch lengths) using the phyloFit program[3]. Summaries of the estimated parameters are given in Figure S1. We call this model the CONS model.

The REV model is the most general model for nucleotide substitution subject to the time-reversibility constraint. This model implies a particular parameterization of the rate matrix for nucleotide substitutions, which has four nucleotide frequencies and five rate parameters. Other parameterizations of the substitution rate matrix could be considered. However, we selected the REV model because of its general applicability and do not expect the results to depend heavily on this choice. Note that for simplicity we use a model that assumes independence between bases. Employing a context dependent model would allow us to easily model substitution patterns over two or three adjacent bases, alleviating the need to remove CpG dinucleotides from the data set. However, a context dependent model might present problems with estimation in this setting. It remains to be shown whether employing a more heavily parameterized model will in fact provide more power to detect human-specific changes.

Our model for nucleotide evolution could be modified to include indels, allowing us to utilize rather than discard information from alignment columns with gaps. We explored the possibility of treating gaps as a fifth character and identified a number of genomic elements with more human-specific indels than expected (data not shown). One serious drawback to this approach, however, is that insertions and deletions often affect

more than one adjacent base, violating the assumption of independence between bases. Consequently, large indels are assigned a higher probability then they deserve. A solution would be to model indel events, rather than indel bases (see ref. 3 for some work on this problem).

S1.3 Likelihood Ratio Test.

LRT statistics. For each region, we compute the likelihood ratio test (LRT) statistic as follows. First, we fit two models to the multiple alignment data for the region. Both are scaled versions of the CONS model, a technique that avoids re-estimation all model parameters on a small amount of data. The null model has a single scale parameter representing a shortening (more conserved) or lengthening (less conserved) of all branches in the CONS tree. The alternative model has an additional parameter for the human branch, which is constrained to be ≥ 1. This extra parameter allows the human branch to be relatively longer (less conserved) than the branches in the rest of the tree. Both models are fit using the phyloFit function (phast library) with the --init-model and --scale-only options. The model with a human rate parameter is fit with the additional option --scale-subtree human:loss. The LRT statistic is the log ratio of the likelihood of the alternative model to that of the null model. Regions are ranked based on the magnitude of the LRT statistic, with larger values indicating more evidence for acceleration in human. The ranking on LRT statistics agrees well with other methods[4].

LRT p-values. It is also of interest to assign a measure of statistical significance to each LRT statistic. We compute empirical p-values by simulation from the CONS model. One million simulated data sets are generated using the phyloBoot program (phast library). These are of variable lengths (median=140, as in the observed data). For each simulated data set, the LRT statistic is computed as above. The distributions of these statistics are similar for different length elements, so we pool all simulated LRT statistics to form a single null distribution. For each observed LRT statistic, the empirical p-value is the proportion of simulated data sets with a larger LRT statistic. Note that the smallest p-value that can be estimated by this method ($1e^{-6}$ here) depends on the number of simulated data sets. For observed LRT statistics that exceed all simulated LRT statistics, we can only say $p < 1e^{-6}$. Computational burden prevents more precise estimation.

S1.4 Multiple comparisons.

Because the genomic regions we study in this paper are on different chromosomes or are separated by significant distance we can assume independence of their nucleotide substitution processes. This assumed independence allows us to employ a simple multiple testing correction throughout this study, the Benjamini & Hochberg False Discovery Rate (FDR) controlling procedure[5], which requires independence or weak dependence between tests. The smallest FDR adjusted p-value that we can compute in the LRT is $4.5e^{-4}$.

S1.5 Filtering.

In order to illustrate the types of erroneous elements that would be found in the list of HARs if we did not perform filtering (Section 4.3), we describe the following elements that were removed from the analysis.

One high probability element, hg17.chr13:22,408,812-22,408,911, has a paralog in chimp and human, but not the rodents. This element is eliminated because we could not determine conclusively (due to gaps in the chimp assembly) which chimp sequence should align to each human sequence.

There are two relatively significant elements that contain multiple adjacent human changes: hg17.chr10:127,180,121-127,180,192 and hg17.chr11:118,305,215-118,305,352. In both cases, recomputing the element ranking without the adjacent changes seriously reduces the overall significance of the element (regardless of which specific base is retained). Hence, we eliminate these elements from further study.

For two elements, hg17.chrX:95,820,769-95,820,993 and hg17.chrX:95,820,618-95,820,729, both of which fall in an intron of the DIAPH2 (O60879) gene, the chimpanzee reads in NCBI in fact agree with the human sequence. This suggests that the chimp whole genome assembly may be incorrect at this position and the substitutions are primate specific, but not human-specific. Furthermore, the macaque sequence in these two elements agrees with human, supporting the chimp reads and not the chimp assembly. We believe that the chimpanzee sequence in the corresponding Contig #300019 is an assembly error, potentially caused by mouse contamination of the chimpanzee library.

One high scoring element, hg17.chr18:74,236,384-74,236,609, is removed based on contradictory findings in our resequencing data. A 4bp deletion in the human genome relative to the chimp and rodent genomes is not found in any of the humans in the PDR panel. Furthermore, all reads in the NCBI trace repository also do not have the deletion. This suggests that it is either a rare mutation or an assembly or read error. Because the apparent human-specific changes in this element are explained by a shift in the alignment due to this questionable indel, we remove the element from the analysis.

S1.6 Background substitution rates based on ENCODE data.

Background substitution rates are estimated using 4-fold degenerate (4d) sites in the ENCODE regions[6] (http://www.genome.gov/10005107), which cover 1% of the human genome. We fit a REV substitution model[1] to 4d sites from all ENCODE regions and from the five ENCODE regions that fall in the last (distal) band of their chromosomes. The rate matrix and GC content parameters were adjusted to correct for known bias in 4d sites using genome-wide estimates from ancestral repeats. These regions have a similar distribution of distances to the chromosome end as the HAR elements, making their 4d sites a suitable data set to estimate background substitution rates near chromosome ends. For each fitted model, we compute the posterior expected value of the number of substitutions on each lineage with the program phyloP with option --subtree (phast library). The background rates in the human-chimp tree are compared to the estimated chimp and human rates in the HAR elements.

S1.7 Does selecting regions based on divergence bias the results of an HKA test?

To test the hypothesis that beginning with high divergence regions might bias the results of HKA-like tests, we conducted a simulation study. We generated 10^6 simulated data sets from a model without selection. Then, we compared the distribution of the HKA p-values for the top N% (N=1,10) to the full distribution. The p-value distributions are not distinguishable, and in particular they have similar sized tails. The false positive rate (*i.e.* the number of $p < 0.05$) is the same in both sets over repeated rounds of the simulation. Hence, we conclude that the use of high divergence regions should not bias our tests for selection.

S1.8 SNP detection bias in dbSNP

In an effort to directly quantify the level of bias in our SNP detection using the dbSNP125, we compared counts of segregating sites in non-overlapping windows of various sizes (5kb-50kb) from the 10 Hapmap resequenced ENCODE regions (*i.e.* an unbiased, non-ascertained dataset) to counts obtained from the dbSNP125 data that we have used here (*i.e.* a dataset of mixed origin and ascertainment). We combine all 10 ENCODE regions and compute Spearman's correlation coefficient across all windows of a fixed size spanning the data set. Windows at three scales were examined: 5kb, 10kb, and 50kb. In each case, a strong correlation was found between the number of SNPs discovered in each dataset (Spearman's ρ values: 50kb $\rho = 0.814$, 10kb $\rho = 0.72$, 5kb $\rho = 0.66$). Thus, although a non-ascertained dataset is not available for the genomic regions surrounding the HAR1-HAR5, the levels of variation detected using dbSNP data should accurately reflect the actual genomic levels of polymorphism on the scales analyzed here.

Because we only utilize numbers of observed polymorphisms, and not their frequencies, our analyses could potentially be robust to certain forms of ascertainment bias[7, 8]. The qualitative insensitivity of our findings to sample size (Section 2.5) suggests that this may be the case. Another potential source of robustness comes from the fact that our hypothesis tests are based on comparisons across genomic windows. Hence, even though the estimate of species divergence time in our coalescent-based approach (Text S1.10) will differ between an unbiased sample and dbSNP data, the probability of the observed configuration of fixed and segregating sites conditional on the estimated divergence time should not be significantly affected.

S1.9 Diversity in HAR1 region compared to Seattle/NIEHS SNPs data

A 6.5Kb region around HAR1 was resequenced in 37 individuals producing a

folded site frequency spectrum consistent with the neutral model (*i.e.* no skew was found)[4]. Twenty-five of the 37 sequenced individuals (10 African Americans and 15 CEPH Caucasians) belong to the panel ("Panel 1") used for resequencing by the Seattle SNPs project (http://pga.gs.washington.edu). In order to evaluate diversity in the HAR1 region relative to genome-wide averages, we computed several population genetic measures for the 6.5Kb HAR1 region and compared these to distributions for those measures in the Seattle SNPs data (178 genes). Only the overlapping 25 individuals were used in the comparison. Since the HAR1 region is non-coding, we performed the analysis for all of the Seattle SNPs sequenced regions and for introns only. Singleton SNPs (present in only 1 individual) as well as insertion and deletion polymorphisms are excluded from the computations.

Estimates of several population genetic parameters for the 6.5Kb HAR1 region are:

- **Number of segregating sites:** $S = 29$,
- **Mutation rate (population scaled):** $\theta_W = 0.0014$, $\theta_\pi = 0.0018$,
- **Tajima's D:** $D = 1.12$.

These values can be compared to the distributions from Seattle SNPs regions. Regardless of whether only introns or the whole sequenced regions are used for the Seattle SNPs data, it appears that the HAR1 region has a high mutation rate (97th percentile for θ_W and 98th percentile for θ_π with introns only). The value of Tajima's D for the HAR1 region is some what high (74th percentile with introns only). These findings suggest that a mutational hot spot could have been at least partially responsible for the rapid evolution in the HAR1 region. The fact that we detect reduced diversity relative to divergence in the HAR1 region (Section 2.5) despite there being a very high level of diversity underscores that fact that divergence is exceptionally high at this locus.

S1.10 Selective sweep analysis using the coalescent.

The speciation model used is the simplest kind, where at a certain time T, a single ancestral species splits into two daughter species, with identical population sizes. The method employs a two-stage approach.

First, we estimate the species divergence time T by simulating coalescent genealogies as follows. Let M be the total number of mutations observed in a sequence sample. These can be divided into mutations occurring since the most recent common ancestor of humans (segregating sites, S) and mutations occurring on the rest of the tree (fixed differences between the species, D). Then, $M = S + D$. Let $x = \{S, D\}$ denote a particular observed sample configuration. Conditional on x, T creates a covariance between S and D. Through simulation, we can therefore evaluate the likelihood of T given data x. That is,

$$lik(T \mid x) \propto Pr(x \mid T, M) \approx \frac{1}{g}\sum_{i=1}^{g} 1(x_i = x)$$

where g, the number of simulations, is appropriately large ($g = 10^6$ here) and $1(\cdot)$ is the indicator function. Simulation out of the coalescent is simple, and it is therefore straightforward to estimate the probability of a sample configuration by generating multiple genealogies, over which we lay down M mutations according to the standard "fixed S" method[9], and then evaluate whether of not each simulated sample configuration is the same as what we observe in our data. As we are now in the summary-likelihood setting[10, 11], we can combine data across k independent loci to obtain a genomic estimate of time since speciation, T', by taking the product of the individual likelihood values such that

$$lik(T') = \prod_{i=1}^{k} lik(T_i \mid x_i).$$

Thus, we can obtain a coalescent-based ML estimate of species divergence time. We perform this estimation using the $k = 4$ observed sample configurations for the 1Mb genomic regions surrounding the four HAR elements (at three scales of analysis: 1kb, 5kb, and 10kb).

Next, because we are interested in selective sweeps, we evaluate the probability of a locus having S or fewer segregating sites conditional on our ML estimate of T and the number of fixed differences at that locus. This probability is evaluated at each locus of interest (at each scale centered upon each of the top four elements) and provides an estimate of the probability of a selective sweep in each region (proportion of 10^5 simulated data sets with S or fewer segregating sites).

As human demographic history is known to be complex, we performed the complete estimation and testing procedure under four different demographic models at each of the three scales of analysis. It should be noted that our results are qualitatively similar across all demographic models. The four models explored were 1) the standard neutral model (constant population size), 2) a recent population expansion where the human population expanded in size from 10^4 to 10^5 starting 1000 generations ago, 3) a more ancient population expansion where the human population grew from 10^4 to 10^5 starting 5000 generations ago, and 4) a model of a population bottleneck followed by subsequent expansion where population size instantaneously decreased from 10^4 to 10^3 5000 generations ago and lasted to 2500 generations ago at which point to population grew to size 10^5.

S1.11 Estimation of the selection coefficient.

The relative rate of substitution for selected mutations to neutral mutations in the Wright-Fisher model is

$$\omega = \frac{f_s}{f_0} \frac{2\gamma}{1 - e^{-2\gamma}},$$

where $\gamma = 2Ns$ is the population scaled selection coefficient, and f_s/f_0 is the ratio of the fraction of mutations that are selected to the fraction that are not. It is typical to assume $f_s/f_0 = 1$. Then, if $\omega = 0.11/0.009 = 12.47$, as we observe in the HAR elements compared to the neutral rate in the last band of chromosome arms, $\gamma = 6.24$. If we use the genome-wide neutral rate $\omega = 0.11/0.0065 = 16.92$ and $\gamma = 8.46$. Because $-2 \leq \gamma \leq 2$ is considered nearly neutral, the selection coefficient would be at least three to four times higher in HAR1 and HAR2 compared to the neutral rate.

S1.12 RNA secondary structure predictions

RNA secondary structure predictions based on a phylogenetic stochastic context free grammar (phylo-SCFG) are available in the EvoFold track of the UCSC genome browser at http://genome.ucsc.edu. These predictions are based on a multiple alignment of 8 vertebrate species. Eighty-eight of the 202 HARs overlap a predicted structure on either the forward or reverse strand (or both). In order to rank these structures based on evidence in the substitution pattern that supports the predicted structure, a score is computed for each structure:

$$S = \#compensatory + 0.25 * \#compatible - 0.5 * \#contradictory.$$

This linear combination of different substitution types was found to perform well on known non-coding RNAs and was used in Pedersen *et al.*[12].

To assess the significance of the observed scores S, shuffling experiments of the HARs extended by 50 nucleotides to both sides are performed as described in Pedersen *et al.*[12]. An empirical p-value for the observed S can be computed as the proportion of the 1000 permuted data sets with a score at least as a large as S. The p-value evaluates how extreme the native structure is given the alignment composition. When there is a structure prediction on both strands, the one with the larger score is used. Twelve of the 88 HARs with predicted structures have unadjusted $p < 0.05$ (Table S4). None is significant at the 0.05 level after FDR adjustment.

S1.13 PhastCons elements containing HAR1-HAR5.

We use the phastCons program (phast library), as described in Siepel *et al.*[2] to predict conserved regions in the 17 species alignments around each primate-rodent conserved block. HAR1-HAR5 each lie in a region that predicted to be highly conserved in the 17 vertebrates (Table S9). These extended HAR regions are between 155bp (HAR2) and 463bp (HAR4) in length. Human acceleration in these five regions is even higher than in HAR1-HAR5, with the human substitution rate varying from 12.8 to 37.5 times the chimp rate. W$\rightarrow$S bias is as high or higher than in HAR1-HAR5, with 75.8% of all human-specific changes from AT to GC nucleotide pairs compared to 8.0% from GC to AT.

S1.14 Biology of the fastest evolving elements.

HAR1-HAR5 all lie in non-coding regions of the human genome that are not currently well characterized. This suggests that they might be involved in the regulation of nearby genes (*e.g.* acting as enhancers). Such regions have been increasingly studied[2, 13, 14], and mounting evidence suggests that highly conserved non-coding elements can regulate expression of nearby developmental transcription factors[15-18]. Interestingly, among the genes nearby these elements, two are known to be involved in human disease and several are expressed primarily in the brain and nervous system. While provocative bioinformatic clues such as these provide some insight, we emphasize that additional experimental work is needed to determine the functions of the HAR elements. We describe what is known about each element individually. All data are available at http://genome.ucsc.edu, unless otherwise noted.

HAR1. The element with the most significant evidence for recent human acceleration overlaps two forward strand mRNAs, BC035016 and BC047717 (both sequenced from human hippocampus), and one reverse strand EST, DB088004 (sequenced from human testis). We describe the forward transcript (named HAR1F) and the overlapping reverse strand transcript (HAR1R) in ref. 4 and show that the entire HAR1 conserved region is transcribed. Secondary structure, compensatory substitutions, and lack of a convincing ORF indicate that HAR1 is part of an RNA gene expressed during neocortical development[4]. The DNA sequences corresponding to the two exons of the *HAR1F* mRNAs align with primates and dog, but not the rodents, indicating that they are evolving much more quickly than the HAR1F conserved region.

HAR2. HAR2 is a CpG island that lies in the eighth intron of the human centaurin gamma 2 (*CENTG2*) gene (Q9UPQ3), which encodes a nuclear protein belonging to a GTPase-activating protein family involved in membrane traffic and actin cytoskeleton dynamics. This element is part of a cluster of non-coding regions conserved between human and frog that lie in a ~750Kb neighborhood of the developmental gene gastrulation brain homeo box 2 (*GBX2*) (P52951). Such highly conserved segments surrounding developmental transcription factors are often enhancers[15-18]. GBX2 is believed to act as a transcription factor for cell pluripotency and differentiation in the embryonic brain. Its homeodomain has homologs in both mouse and chicken. In mouse, GBX2 is a hindbrain marker. The element we discovered lies 302.6Kb away from the start of *GBX2*, compared to 371.3kb for the start of *CENTG2* itself. HAR2 could also be a close range regulator of the transcript represented by the 3' polyadenylated mRNA AK074478, obtained from human lung tissue, which has 99.7% sequence identity (only sequence match in genome by the BLAT program[19]) with a region 2,058bp downstream of HAR2 (also in intron eight of CENTG2).

HAR3. HAR3 lies in the ninth intron of the alternatively-spliced mitotic checkpoint protein isoform MAD1a (*MAD1L1*) gene (Q9Y6D9). Mutations in *MAD1L1* are associated with chromosomal instability and may play a pathogenic role in human cancers[20]. *NUDT1* (P36639) (involved in the sanitization of nucleotide pools by

hydrolyzing oxidized purine nucleoside triphosphates) and *FTSJ2* (Q9UI43) (a putative RNA methyltransferase) are 75.8Kb and 85.4Kb upstream, respectively, and could be regulated by HAR3. In addition, we predict a putative homolog of the *SLIT* genes (key players in axon guidance during neural development) at hg17.chr7:1,557,474-1,559,960, about 410kB away from HAR3. Conservation between human and chicken suggests that HAR3 could potentially be a regulator of any of these genes.

HAR4. HAR4 lies Kb upstream of the AT-binding transcription factor (*ATBF1*) gene (Q15911), which is a transcriptional activator known to bind to an enhancer of the *AFP* gene (P02771) on chromosome 4. *AFP* encodes alpha-fetoprotein, a major serum protein, produced primarily during fetal life and thought to be the fetal counterpart of serum albumin. The level of AFP in amniotic fluid is used to measure renal loss of protein to screen for spina bifida and anencephaly. AFP expression in adults can be associated with hepatoma and teratoma. Conservation between human and chicken suggests that HAR4 could potentially be a regulator of ATBF1.

HAR5. The longest of the top four HAR elements (348bp) is a CpG island that is conserved between human and fish. HAR5 lies in the eighth intron of the cytoplasmic serine-threonine kinase WNK1 (*PRKWNK1*) gene (Q9H4A3), which is associated with familial hypertension. A novel human disease gene, *HSN2* (Q6IFS5), has recently been documented about 2.5kb downstream of HAR5 in intron eight of the *PRKWNK1* gene[21]. It is possible that HAR5 is involved in the regulation of the single exon gene *HSN2*, which causes the autosomal recessive disorder hereditary sensory and autonomic neuropathy (HSAN) type II. Interestingly, two non-human ESTs (cow: CB465313 and mouse: BB660048) map to the location of HAR5, indicating that it may be transcribed.

S1.15 HAR5 polymorphism.

Resequencing of HAR5 showed that all but one of the human-specific changes appear to be fixed in the human population. The site hg17.chr12:844,587 is polymorphic. Seventy percent of individuals are homozygous G (matching the human assembly), 10% are homozygous C (which is the base in the assemblies of all currently available mammals, chicken and frog), and 20% are heterozygous GC. These data suggest that the G in the human genome assembly is the derived allele, which has almost fixed in the human population. Interestingly, two additional human polymorphisms were also found. A polymorphic base was observed at hg17.chr12:844,665, which is homozygous C in 44%, homozygous T in 17%, and heterozygous C/T in 39% of the PDR panel. If the more common C allele had been in the human assembly, this would have been a ninth human-specific difference (since chimp, mouse and rat assemblies are all T). This base is directly next to another human-specific difference (hg17.chr12:844,666). A polymorphic 1bp deletion was observed between hg17.chr12:844617 and hg17.chr12:844618, with 39% of the panel having a C (as do the assemblies of all currently available mammals, chicken and frog) and 61% having a deletion (as does the human assembly). Indels are not included in our statistical analysis, but this example is noted here as further evidence of recent evolutionary activity in this region.

Publicly available polymorphism data support the findings from resequencing. Of the top 5 HAR elements, only HAR5 contains publicly annotated polymorphisms: rs11611231 at hg17.chr12:844,587, rs7300829 at hg17.chr12:844,665, and rs11441897 at hg17.chr12:844,617-844,618. All three were detected in the PDR panel.

References

1. Tavare, S. (ed.) Some probabilistic and statistical problems in the analysis of DNA sequences (American Mathematical Society, 1986).

2. Siepel, A. et al. Evolutionarily conserved elements in vertebrate, insect, worm, and yeast genomes. Genome Res 15, 1034-50 (2005).

3. Siepel A, P. K., Haussler D. New methods for detecting lineage-specific selection. Proceedings of the 10th International Conference on Research in Computational Molecular Biology (RECOMB), 190-205 (2006).

4. Pollard KS, S. S., Lambert N, Coppens S, Pedersen J, et al. An RNA gene expressed during cortical development evolved rapidly in humans. Nature Online Publication (2006).

5. Benjamini, Y. & Hochberg, Y. Controlling the False Discovery Rate - a Practical and Powerful Approach to Multiple Testing. Journal of the Royal Statistical Society Series B-Methodological 57, 289-300 (1995).

6. ENCODEConsortium. The ENCODE (ENCyclopedia Of DNA Elements) Project. Science 306, 636-40 (2004).

7. Wakeley J, N. R., Liu-Cordero S, Ardlie K. The discovery of single-nucleotide polymorphisms–and inferences about human demographic history. American Journal of Human Genetics 69, 1332-1347 (2001).

8. Clark A, H. M., Bustamante C, Williamson S, Nielsen R. Ascertainment bias in studies of human genome-wide polymorphism. Genome Research 15, 1496-1502 (2005).

9. Hudson, R. Gene genealogies and the coalescent process. . Oxford Surveys in Evolutionary Biology 7, 1-44 (1990).

10. Weiss G, v. H. A. Inference of population history using a likelihood approach. Genetics 149, 1539-1546 (1998).

11. Wall, J. A comparison of estimators of the population recombination rate. Molecular Biology and Evolution 17, 156-163 (2000).

12. Pedersen, J. S. et al. Identification and Classification of Conserved RNA Secondary Structures in the Human Genome. PLOS Computational Biology to appear? (2006).

13. Bejerano, G. et al. Ultraconserved elements in the human genome. Science 304, 1321-5 (2004).

14. Dermitzakis E, R. A., Antonarakis S. Conserved non-genic sequences - an unexpected feature of mammalian genomes. Nature Review Genetics 6, 151-157 (2005).

15. **Nobrega, M. A., Ovcharenko, I., Afzal, V. & Rubin, E. M. Scanning human gene deserts for long-range enhancers. Science 302, 413 (2003).**
16. **Shin J, P. J., Ovcharenko I, Ronco A, Moore R, et al. Human zebrafish non-coding conserved elements act in vivo to regulate transcription. Nucleic Acids Research 33, 5437–5445 (2005).**
17. **Woolfe A, G. M., Goode D, Snell P, McEwen G, et al. Highly conserved non-coding sequences are associated with vertebrate development. PLoS Biology 3, e7 (2005).**
18. **Bejerano G, L. C., Ahituv N, King B, Siepel A, et al. A distal enhancer and an ultraconserved exon are derived from a novel retroposon. Nature 441, 87-90 (2006).**
19. **Kent, W. J. BLAT--the BLAST-like alignment tool. Genome Res 12, 656-64 (2002).**
20. **Tsukasaki K, M. C., Greenspun E, Eshaghian S, Kawabata H, et al. Mutations in the mitotic check point gene, MAD1L1, in human cancers. Oncogene 20, 3301-3305 (2001).**
21. **Lafreniere R, M. M., Dube MP, MacFarlane J, O'Driscoll M, et al. Identification of a novel gene (HSN2) causing hereditary sensory and autonomic neuropathy type ii through the study of Canadian genetic isolates. American Journal of Human Genetics 74 (2004).**
