# Supplementary material for: Forces Shaping the Fastest Evolving Regions in the Human Genome
Source: PLoS Genet. 2006 Oct 13;2(10):e168. doi: 10.1371/journal.pgen.0020168 (PMC1599772; doi:10.1371/journal.pgen.0020168)
Supplement: Supplementary file 2 [file pgen.0020168.sg002.pdf]

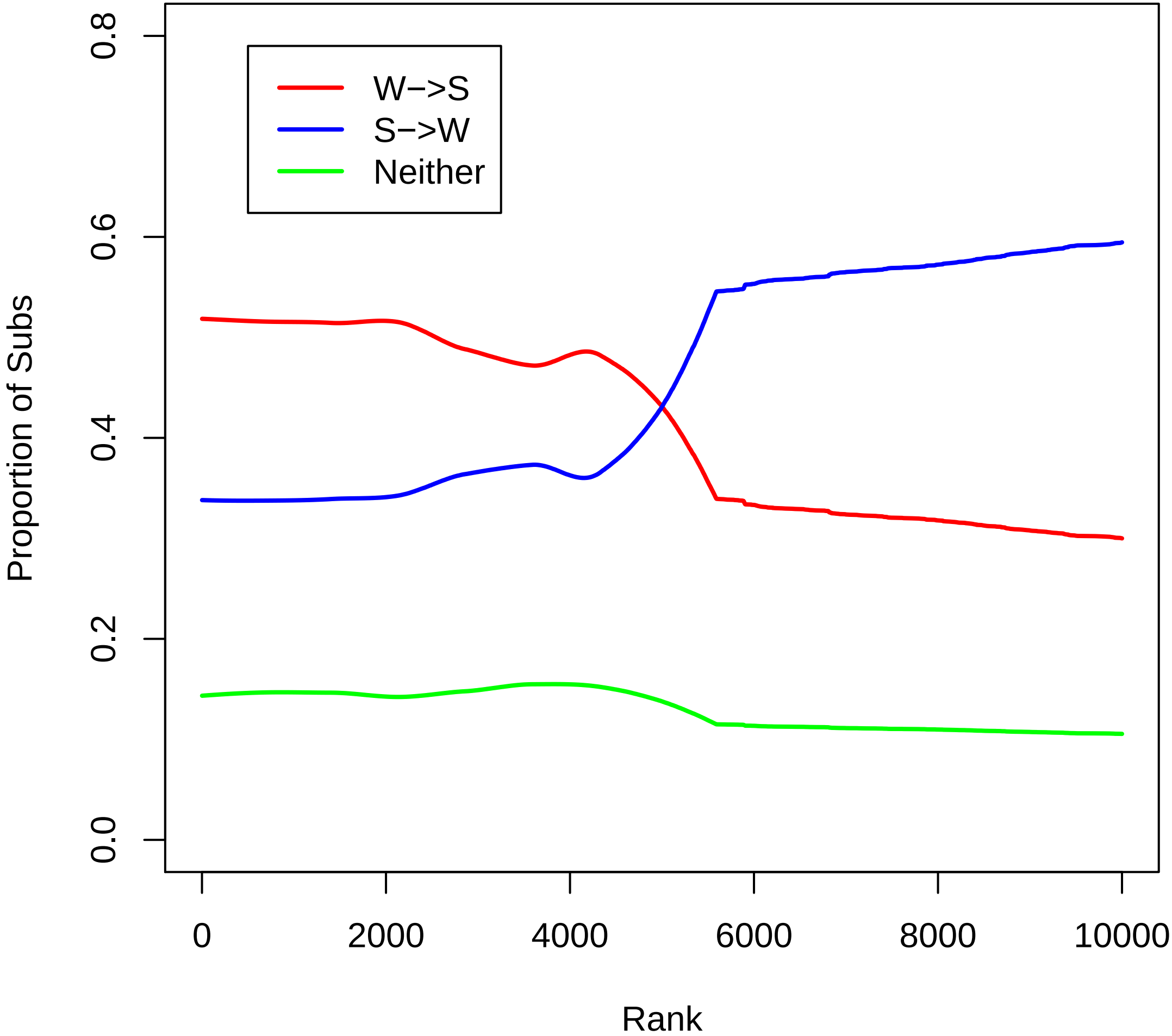

W−>S
S−>W
Neither
Proportion of Subs
0.0
0.2
0.4
0.6
0.8
0
2000
4000
6000
8000
10000
Rank
