# Supplementary material for: Forces Shaping the Fastest Evolving Regions in the Human Genome
Source: PLoS Genet. 2006 Oct 13;2(10):e168. doi: 10.1371/journal.pgen.0020168 (PMC1599772; doi:10.1371/journal.pgen.0020168)
Supplement: Supplementary file 3 [file pgen.0020168.sg003.pdf]

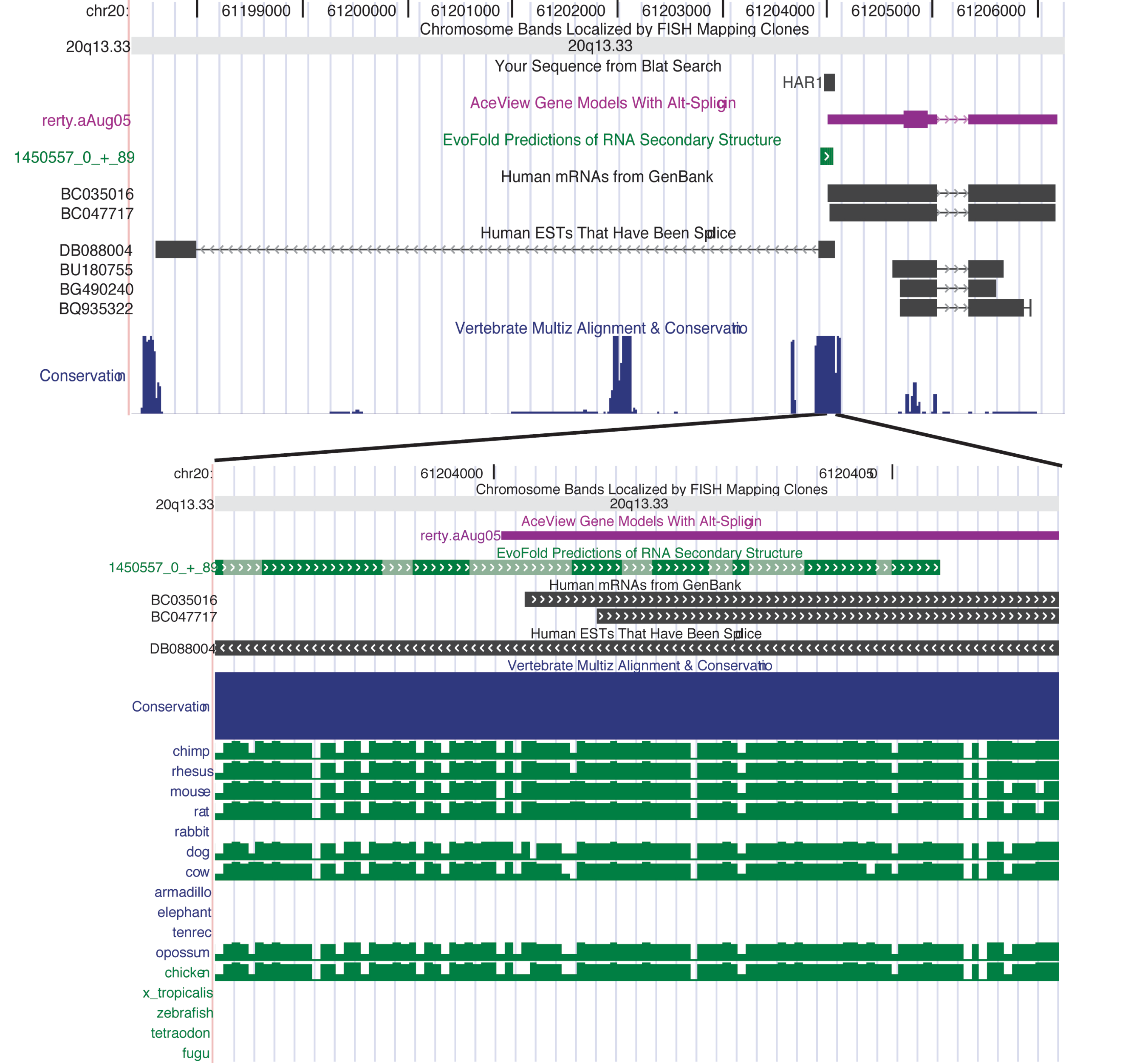

chr20:
61199000
61200000
61201000
61202000
61203000
61204000
61205000
61206000
Chromosome Bands Localized by FISH Mapping Clones
20q13.33
20q13.33
Your Sequence from Blat Search
HAR1
AceView Gene Models With Alt-Splicing
rerty.aAug05
EvoFold Predictions of RNA Secondary Structure
1450557_0_+_89
Human mRNAs from GenBank
BC035016
BC047717
Human ESTs That Have Been Spliced
DB088004
BU180755
BG490240
BQ935322
Vertebrate Multiz Alignment & Conservation
Conservation
chr20:
61204000
61204050
Chromosome Bands Localized by FISH Mapping Clones
20q13.33
20q13.33
AceView Gene Models With Alt-Splicing
rerty.aAug05
EvoFold Predictions of RNA Secondary Structure
1450557_0_+_89
Human mRNAs from GenBank
BC035016
BC047717
Human ESTs That Have Been Spliced
DB088004
Vertebrate Multiz Alignment & Conservation
Conservation
chimp
rhesus
mouse
rat
rabbit
dog
cow
armadillo
elephant
tenrec
opossum
chicken
x_tropicalis
zebrafish
tetraodon
fugu
