# Supplementary material for: Forces Shaping the Fastest Evolving Regions in the Human Genome
Source: PLoS Genet. 2006 Oct 13;2(10):e168. doi: 10.1371/journal.pgen.0020168 (PMC1599772; doi:10.1371/journal.pgen.0020168)
Supplement: Supplementary file 4 [file pgen.0020168.sg004.pdf]

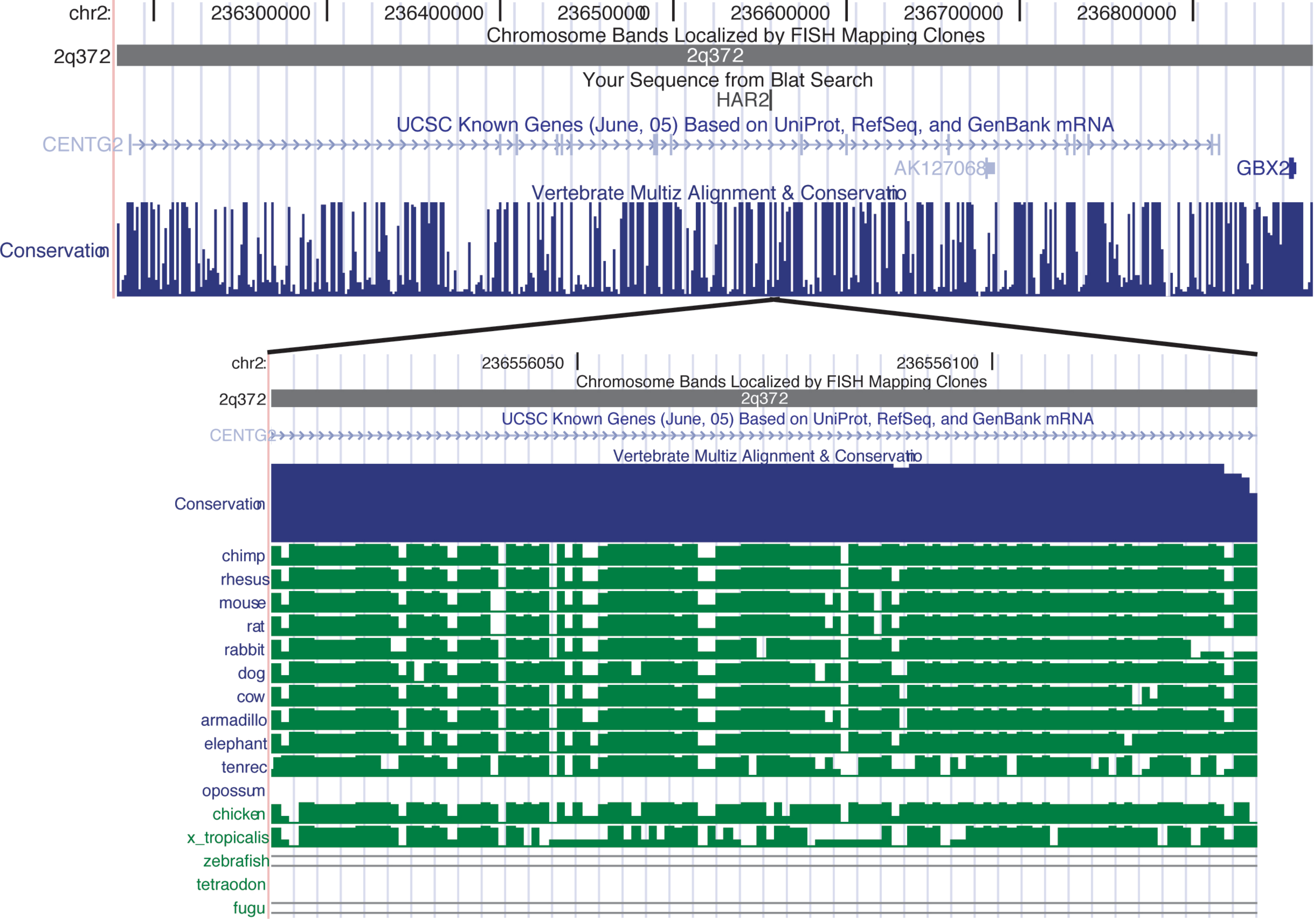

chr2:
236300000
236400000
236500000
236600000
236700000
236800000
Chromosome Bands Localized by FISH Mapping Clones
2q372
2q372
Your Sequence from Blat Search
HAR2
UCSC Known Genes (June, 05) Based on UniProt, RefSeq, and GenBank mRNA
CENTG2
AK127068
GBX2
Vertebrate Multiz Alignment & Conservation
Conservation
chr2:
236556050
236556100
Chromosome Bands Localized by FISH Mapping Clones
2q372
2q372
UCSC Known Genes (June, 05) Based on UniProt, RefSeq, and GenBank mRNA
CENTG2
Vertebrate Multiz Alignment & Conservation
Conservation
chimp
rhesus
mouse
rat
rabbit
dog
cow
armadillo
elephant
tenrec
opossum
chicken
x_tropicalis
zebrafish
tetraodon
fugu
