# Supplementary material for: Forces Shaping the Fastest Evolving Regions in the Human Genome
Source: PLoS Genet. 2006 Oct 13;2(10):e168. doi: 10.1371/journal.pgen.0020168 (PMC1599772; doi:10.1371/journal.pgen.0020168)
Supplement: Supplementary file 5 [file pgen.0020168.sg005.pdf]

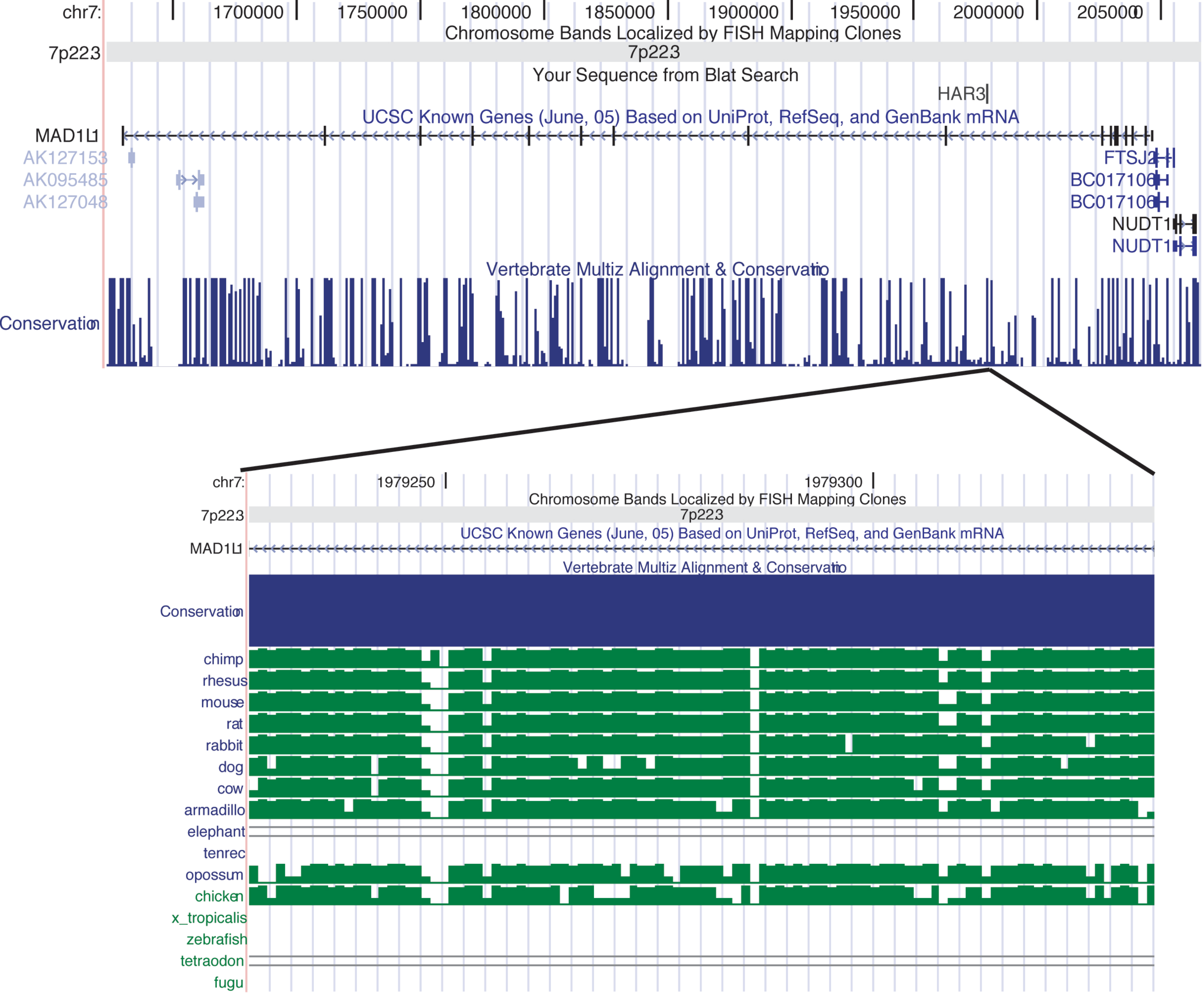
chr7:
1700000
1750000
1800000
1850000
1900000
1950000
2000000
2050000
Chromosome Bands Localized by FISH Mapping Clones
7p22.3
7p22.3
Your Sequence from Blat Search
HAR3
UCSC Known Genes (June, 05) Based on UniProt, RefSeq, and GenBank mRNA
MAD1L1
AK127153
AK095485
AK127048
FTSJ2
BC017106
BC017106
NUDT1
NUDT1
Vertebrate Multiz Alignment & Conservation
Conservation
chr7:
1979250
1979300
Chromosome Bands Localized by FISH Mapping Clones
7p22.3
7p22.3
UCSC Known Genes (June, 05) Based on UniProt, RefSeq, and GenBank mRNA
MAD1L1
Vertebrate Multiz Alignment & Conservation
Conservation
chimp
rhesus
mouse
rat
rabbit
dog
cow
armadillo
elephant
tenrec
opossum
chicken
x_tropicalis
zebrafish
tetraodon
fugu
