# Supplementary material for: Forces Shaping the Fastest Evolving Regions in the Human Genome
Source: PLoS Genet. 2006 Oct 13;2(10):e168. doi: 10.1371/journal.pgen.0020168 (PMC1599772; doi:10.1371/journal.pgen.0020168)
Supplement: Supplementary file 6 [file pgen.0020168.sg006.pdf]

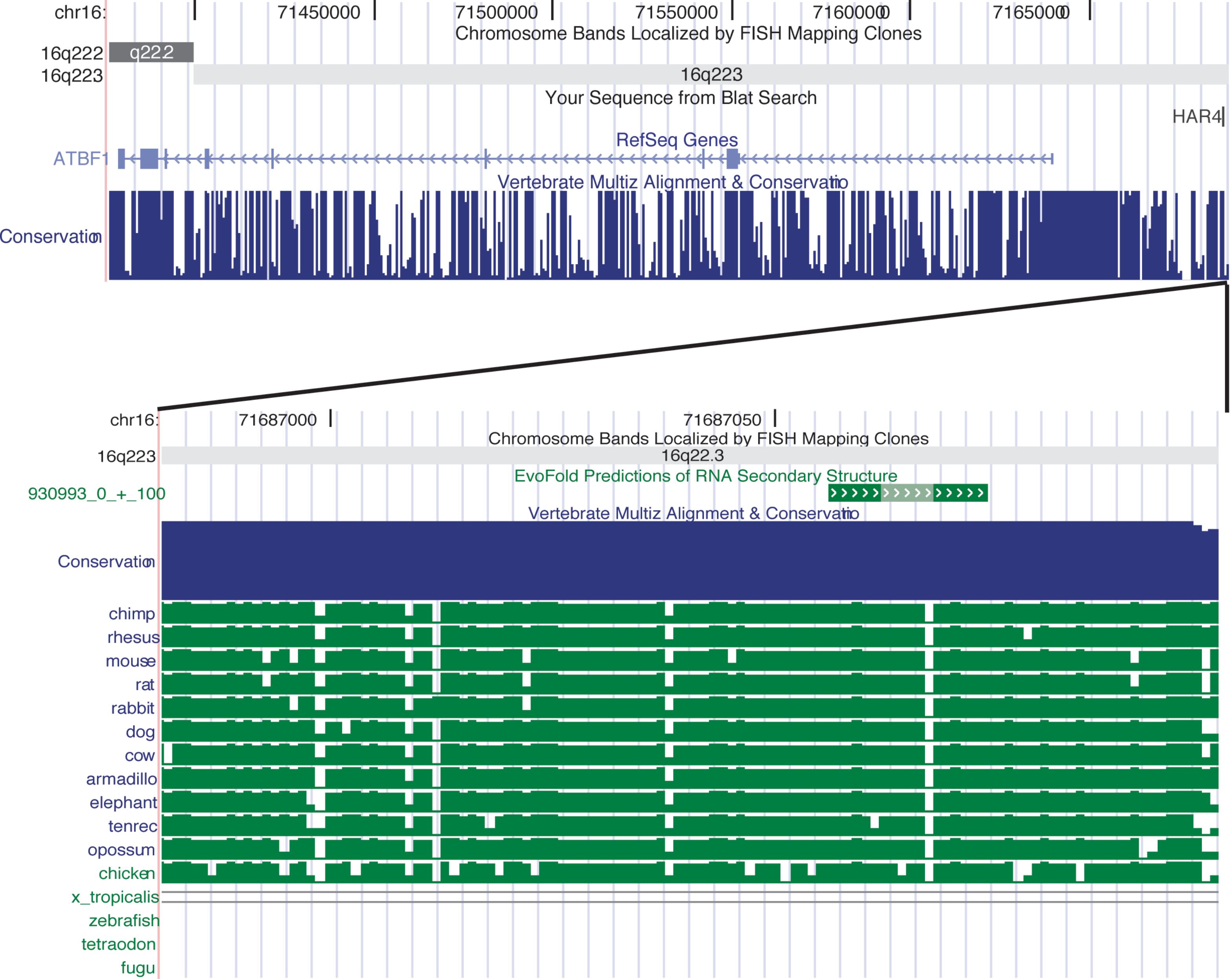

chr16:
71450000
71500000
71550000
71600000
71650000
Chromosome Bands Localized by FISH Mapping Clones
16q222
q22.2
16q223
16q223
Your Sequence from Blat Search
HAR4
RefSeq Genes
ATBF1
Vertebrate Multiz Alignment & Conservation
Conservation
chr16:
71687000
71687050
Chromosome Bands Localized by FISH Mapping Clones
16q223
16q22.3
EvoFold Predictions of RNA Secondary Structure
930993_0_+_100
Vertebrate Multiz Alignment & Conservation
Conservation
chimp
rhesus
mouse
rat
rabbit
dog
cow
armadillo
elephant
tenrec
opossum
chicken
x_tropicalis
zebrafish
tetraodon
fugu
