# Supplementary material for: Forces Shaping the Fastest Evolving Regions in the Human Genome
Source: PLoS Genet. 2006 Oct 13;2(10):e168. doi: 10.1371/journal.pgen.0020168 (PMC1599772; doi:10.1371/journal.pgen.0020168)
Supplement: Supplementary file 7 [file pgen.0020168.sg007.pdf]

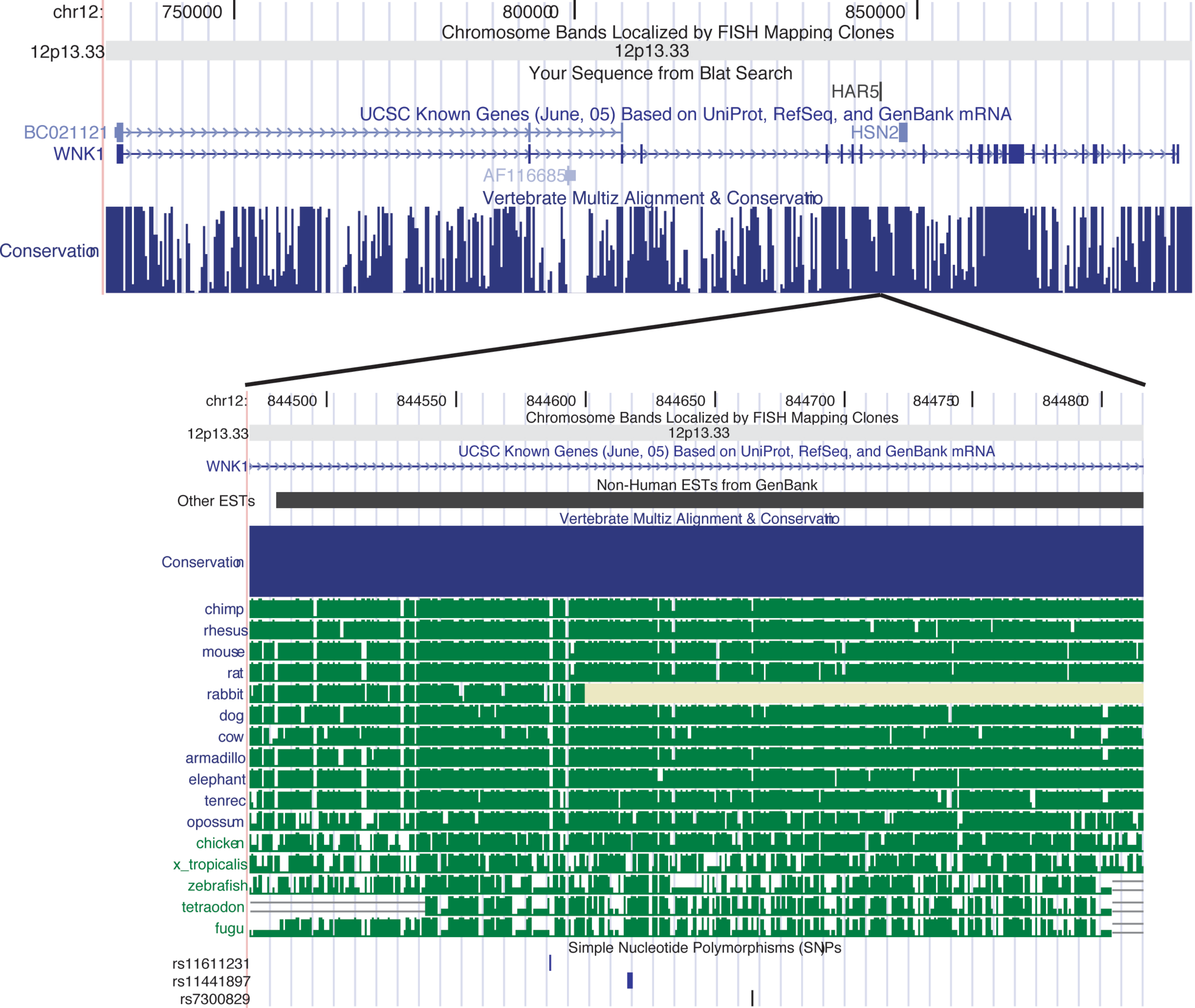
chr12:
750000
800000
850000
Chromosome Bands Localized by FISH Mapping Clones
12p13.33
12p13.33
Your Sequence from Blat Search
HAR5
UCSC Known Genes (June, 05) Based on UniProt, RefSeq, and GenBank mRNA
BC021121
HSN2
WNK1
AF116685
Vertebrate Multiz Alignment & Conservation
Conservation
chr12:
844500
844550
844600
844650
844700
844750
844800
Chromosome Bands Localized by FISH Mapping Clones
12p13.33
12p13.33
UCSC Known Genes (June, 05) Based on UniProt, RefSeq, and GenBank mRNA
WNK1
Non-Human ESTs from GenBank
Other ESTs
Vertebrate Multiz Alignment & Conservation
Conservation
chimp
rhesus
mouse
rat
rabbit
dog
cow
armadillo
elephant
tenrec
opossum
chicken
x_tropicalis
zebrafish
tetraodon
fugu
Simple Nucleotide Polymorphisms (SNPs)
rs11611231
rs11441897
rs7300829
