# Supplementary material for: Forces Shaping the Fastest Evolving Regions in the Human Genome
Source: PLoS Genet. 2006 Oct 13;2(10):e168. doi: 10.1371/journal.pgen.0020168 (PMC1599772; doi:10.1371/journal.pgen.0020168)
Supplement: Supplementary file 8 [file pgen.0020168.sg008.pdf]

HAR1

```
assembly tGtcaGctgaaatGatGggCgtagacGcaCgtcagcGgCggaaatGgtttctatcaaaatGaaagtGtttagagattttcctcaaGtttcaaatGaGgcgaatccc
human    tGtcaGctgaaatGatGggCgtagacGcaCgtcagcGgCggaaatGgtttctatcaaaatGaaagtGtttagagattttcctcaaGtttcaaatGaGgcgaatccc
chimp    tAtcaActgaaatTatAggTgtagacAcaTgtcagcAgTggaaatAgtttctatcaaaatTaaagtAtttagagattttcctcaaAtttcaaatTaTgcgaatccc
gorilla  tAtcaActgaaatTatAggTgtagacAcaTgtcagcAgTggaaatAgtttctatcaaaatTaaagtAtttagagattttcctcaaAtttcaaatTaTgcgaatccc
orang    tAtcaActgaaatTatAggTgtagacAcaTgtcagcAgTggaaatAgtttctatcaaaatTaaagtAtttagagattttcctcaaAtttcaaatTaTgcgaatccc
macaque  tAtcaGctgaaatTatAggTgtagacAcaTgtcagcAgTggaaatAgtttctatcaaaatTaaagtAtttagagattttcctcaaAtttcaaatTaTgcgaatccc
```

HAR2

```
assembly ttCcgcaaatagggcaCccacaGtaacaCgtgtgGcGcCGaccccgccgtgcGCaatcggggctttataCgattgCtggagatgctgacagctcaattaaagtgta
human    ttCcgcaaatagggcaCccacaGtaacaCgtgtgGcGcCGaccccgccgtgcGCaatcggggctttataCgattgCtggagatgctgacagctcaattaaagtgta
chimp    ttTcgcaaatagggcaTccacaAtaacaAgtgtgTcAcTAaccccgccgtgcATaatcggggctttataGgattgCtggagatgctgacagctcaattaaagtgta
gorilla  ttTcgcaaatagggcaTccacaAtaacaAgtgtgTcAcTAaccccgccgtgcATaatcggggctttataGgattgCtggagatgctgacagctcaattaaagtgta
orang    ttTcgcaaatagggcaTccacaAtaacaAgtgtgTcAcTAaccccgccgtgcATaatcggggctttataGgattgCtggagatgctgacagctcaattaaagtgta
macaque  ttTcgcaaatagggcaTccacaAtaacaAgtgtgTcAcTAaccccgccgtgcATaatcggggctttataGgattgTtggagatgctgacagctcaattaaagtgta
spider   ttTcgcaaatagggcaTccacaAtaacaAgtgtgTcAcTAaccccgccgtgcATaatcggggctttataGgattgTtggagatgctgacagctcaattaaagtgta

assembly acccttTgtGccc
human    acccttTgtGccc
chimp    acccttTgtAccc
gorilla  acccttTgtAccc
orang    acccttAgtAccc
macaque  acccttTgtAccc
spider   acccttTgtAccc
```

HAR3

```
assembly ttctcccagctcaggaaggatCtCaaggGcttcaaagcagcctgggttccaattaagccCgtgtgaccaccccagggatgcCagaaCattcctgattaacaagagg
human    ttctcccagctcaggaaggatCtCaaggGcttcaaagcagcctgggttccaattaagccCgtgtgaccaccccagggatgcCagaaCattcctgattaacaagagg
chimp    ttctcccagctcaggaaggatTtAaaggActtcaaagcagcctgggttccaattaagccAgtgtgaccaccccagggatgcTagaaTattcctgattaacaagagg
orang    ttctcccagctcaggaaggatTtAaaggActtcaaagcagcctgggttccaattaagccAgtgtgaccaccccagggatgcTagaaTattcctgattaacaagagg
macaque  ttctcccagctcaggaaggatTAAaaggActtcaaagcagcctgggttccaattaagccAgtgtgaccaccccagggatgcTagaaTattcctgattaacaagagg
```

HAR4

```
assembly gcagcttttgtgtcagaccTtacgtgaaaGtaCgcagtaagctgggtaatttatttacTtttt-ggtgattatttttttatgattttatCatcaTattcaAtgattc
human    gcagcttttgtgtcagaccTtacgtgaaaGtaCgcagtaagctgggtaatttatttacTtttt-ggtgattatttttttatgattttatCatcaTattcaAtgattc
chimp    gcagcttttgtgtcagaccCtacgtgaaaAtaAgcagtaagctgggtaatttatttacCtttt-ggtgattatttttttatgattttatGatcaTattcaAtgattc
gorilla  gcagcttttgtgtcagaccCtacgtgaaaAtaAgcagtaagctgggtaatttatttacCtttt-ggtgattatttttttatgattttatGatcaTattcaAtgattc
orang    gcagctttttgtcagaccCtacgtgaaaAtaAgcagtaagctgggtaatttatttacCtttt-ggtgattatttttttatgattttatGatcaTattcaAtgattc
macaque  gcagcttttgtgtcagaccCtacgtgaaaAtaAgcagtaagctgggtaatttatttacCtttt-ggtgattatttttttatgattttatGatcaTattcaGtgattc
spider   gcagcttttgtgtcagaccCtacgtgaaaAtaAgcagtaagctgggtaatttatttacCttttggtgattatttttttatgattttatTatcaTattcaGtgattc
lemur    gcagcttttgtgtcagaccCtacgtgaaaAtaAgcagtaagctgggtaatttatttacCtttt-ggtgattatttttttatgattttatGatcaGattcaAtgattc

assembly ataaacattggcctg
human    ataaacattggcctg
chimp    ataaacattggcctg
gorilla  ataaacattggcctg
orang    ataaacattggcctg
macaque  ataaacattggcctg
spider   ataaacattggcccg
lemur    ataaacattggcctg
```

HAR5

```
assembly tgtacttttgtttattttgttttcaCagtactgtgtttttcatgtgtgtgtTtGttTtGtGttGagcctcgtcgtggccgtagcatgtcggtttgtgttccc-atc
human    tgtacttttgtttattttgttttcaCagtactgtgtttttcatgtgtgtgtTtGttTtGtGttGagcctcgtcgtggccgtagcatgtcggtttgtgttccc-atc
chimp    tgtacttttgtttattttgttttcaAagtactgtgtttttcatgtgtgtgtTtGttTtCtGttTagcctcgtcgtggccgtagcatgtcggtttgtgttcccCatc
gorilla  tgtacttttgtttattttgttttcaAagtactgtgtttttcatgtgtgtgtTtGttTtCtGttTagcctcgtcgtggccgtagcatgtcggtttgtgttcccCatc
orang    tgtacttttgtttattttgttttcaAagtactgtgtttttcatgtgtgtgt-t-ttCtGt-tt-agcctcgtcgtggccgtagcatgtcggtttgtgttcccCatc
macaque  tgtacttttgtttattttgttttcaAagtactgtgtttttcatgtgtgtgtTtGttTtCtGttTagcctcgtcgtggccgtagcatgtcggtttgtgttcccCatc

assembly tttctgctgttGcctctGtgtcccgcatctctcccagtgctc-ttccaccccAccGccagtActgtctgcacctctttctccttccctcctTcggactgcccc-ga
human    tttctgctgttGcctctGtgtcccgcatctctcccagtgctc-ttccaccccAccGccagtActgtctgcacctctttctccttccctcctCcggactgcccc-ga
chimp    tttctgctgttCcctctCtgtcccgcatctctcccagtgctcCttccaccccAccAccagtGctgtctgcacctctttctccttccctcctTcggactgccccTga
gorilla  tttctgctgttCcctctCtgtcccgcatctctcccagtgctcCttccaccccGccAccagtGctgtctgcacctctttctccttccctcctTcggactgccccTga
orang    tttctgctgttCcctctCtgtcccgcatctctcccagtgctcCttccaccccAccAccagtGctgtctgcacctctttctccttccctcctTcggactgccccTga
macaque  tttctgctgttCcctctCtgtcccgcatctctcccagtgctcCttccaccccAccAccagtGctgtctgcacctctttctccttccctcctTcggactgccccTga

assembly ggaaacttttgccGaaaagctttctaaagcattggagagtgtcctgcctatgcactctgcctctcagCgcaagcaccgacgctccagcctgccttccctctttgtc
human    ggaaacttttgccGaaaagctttctaaagcattggagagtgtcctgcctatgcactctgcctctcagCgcaagcaccgacgctccagcctgccttccctctttgtc
chimp    ggaaacttttgccGaaaagctttctaaagcattggagagtgtcctgcctatgcactctgcctctcagCgcaagcaccgacgctccagcctgccttccctctttgtc
gorilla  ggaaacttttgccGaaaagctttctaaagcattggagagtgtcctgcctatgcactctgcctctcagCgcaagcaccgacgctccagcctgccttccctctttgtc
orang    ggaaacttttgccAaaaagctttctaaagcattggagagtgtcctgcctatgcactctgcctctcagTgcaagcaccgacgctccagcctgccttccctctttgtc
macaque  ggaaacttttgccGaaaagctttctaaagcattggagagtgtcctgcctatgcactctgcctctcagCgcaagcaccgacgctccagcctgccttccctctttgtc

assembly agtactgtatgtaactgtaaacttctgacaa
human    agtactgtatgtaactgtaaacttctgacaa
chimp    agtactgtatgtaactgtaaacttctgacaa
gorilla  agtactgtatgtaactgtaaacttctgacaa
orang    agtactgtatgtaactgtaaacttctgacaa
macaque  agtactgtatgtaactgtaaacttctgacaa
```
