# Supplementary material for: Forces Shaping the Fastest Evolving Regions in the Human Genome
Source: PLoS Genet. 2006 Oct 13;2(10):e168. doi: 10.1371/journal.pgen.0020168 (PMC1599772; doi:10.1371/journal.pgen.0020168)
Supplement: Supplementary file 9 [file pgen.0020168.st001.pdf]

	**Length**		
**Percent ID**	**≥ 50 bp**	**≥ 100 bp**	**≥ 200 bp**
≥ 90%	383,576 (79,248)	137,396 (41,378)	40,644 (18,374)
≥ 92%	299,143 (56,749)	94,605 (26,727)	26,407 (11,215)
≥ 94%	217,428 (37,879)	60,234 (16,051)	16,051 (6,324)
≥ 96%	143,912 (22,683)	34,945 (8,370)	8,707 (3,052)
≥ 98%	84,182 (11,510)	17,484 (3,518)	3,678 (1,050)
100%	38,897 (5,068)	5,131 (700)	466 (79)

Table S1. Percent identity in chimp-rodent alignments. Counts of the number of blocks in multiple alignments of the chimpanzee, mouse, and rat genomes that are a given length and whose nucleotide sequences are a given percent identical (across all three species). Only nucleotides (A,C,T,G), and not indels nor missing data, count as identical. The number in parentheses gives the count of these blocks that contain at least one alignment column where chimpanzee, mouse, and rat have an identical nucleotide and human has a different one (*human diff*). The 466 blocks ≥ 200 bp and 100% identical are analogous to (but not identical to) the ultraconserved elements described in ref. 32, which used the human, mouse and rat genomes.
