# Supplementary material for: Forces Shaping the Fastest Evolving Regions in the Human Genome
Source: PLoS Genet. 2006 Oct 13;2(10):e168. doi: 10.1371/journal.pgen.0020168 (PMC1599772; doi:10.1371/journal.pgen.0020168)
Supplement: Supplementary file 10 [file pgen.0020168.st002.pdf]

	Number present in Species	Mean Percent Identical
**Dog**	34194 (99.1%)	96.8%
**Chicken**	25373 (73.6%)	85.7%
**Frog**	18712 (54.2%)	77.7%
**Fish**	12150 (35.2%)	73.3%
**All four species**	10152 (29.4%)	86.8%

Table S2. Conservation in additional species. Conservation patterns of 34,497 primate-rodent conserved regions among more distantly related vertebrates. Mean percent identical gives the average proportion of bases that match human (hg17, build 35) among the regions that are present in that species. Average percent identity with human is 86.8%, which is very high compared to typical orthologously alignable DNA (*e.g.* 67% in mouse[34] and 60% in fish[32]).
