# Supplementary material for: Forces Shaping the Fastest Evolving Regions in the Human Genome
Source: PLoS Genet. 2006 Oct 13;2(10):e168. doi: 10.1371/journal.pgen.0020168 (PMC1599772; doi:10.1371/journal.pgen.0020168)
Supplement: Supplementary file 11 [file pgen.0020168.st003.pdf]

	**Number of human diffs**					
	**> 0**	**> 1**	**> 2**	**> 3**	**> 4**	**> 5**
**Number of Blocks**	8,189	1,663	363	75	16	5

Table S3. Distribution of human-specific changes in conserved blocks. Counts of the number of conserved blocks (out of 34,498) with more than 0-5 human diffs. Human diffs are sites where the chimp, mouse and rat bases are identical, but the human base is different (Text S1.1).
