# Supplementary material for: Forces Shaping the Fastest Evolving Regions in the Human Genome
Source: PLoS Genet. 2006 Oct 13;2(10):e168. doi: 10.1371/journal.pgen.0020168 (PMC1599772; doi:10.1371/journal.pgen.0020168)
Supplement: Supplementary file 12 [file pgen.0020168.st004.pdf]

HAR	Observed score *(S)*	*p*-value
HAR97	2.50	0.001
HAR1	5.50	0.005
HAR188	1.25	0.007
HAR17	1.25	0.011
HAR33	0.50	0.020
HAR155	0.75	0.022
HAR41	0.25	0.024
HAR73	0.75	0.030
HAR27	0.50	0.035
HAR69	1.25	0.037
HAR78	0.50	0.041
HAR190	0.25	0.042

Table S4. HARs with significant RNA secondary structure. Observed Score (S) is a linear combination of counts of different types of substitutions (Text S1.12). P-values are computed empirically by shuffling the columns of the multiple alignment as described in Pedersen *et al.*[37] (not adjusted for multiple comparisons).
