# Supplementary material for: Forces Shaping the Fastest Evolving Regions in the Human Genome
Source: PLoS Genet. 2006 Oct 13;2(10):e168. doi: 10.1371/journal.pgen.0020168 (PMC1599772; doi:10.1371/journal.pgen.0020168)
Supplement: Supplementary file 13 [file pgen.0020168.st005.pdf]

	**HAR1**	**HAR2**	**HAR3**	**HAR4**	**HAR5**	**Total**
Substitution Rates						
**Human:Chimp**	3.56	12.25	12.93	17.33	10.21	7.24
**Human:(Chimp-Mouse)**	76.45	50.32	46.08	20.17	9.27	25.87

Table S5. Comparison of estimated substitution rates in HAR1-HAR5. Substitution rates are posterior expected value of the number of substitutions using the method described in Siepel *et al.*[38] with the CONS model as prior. The human:chimp ratio is the estimated number of substitutions per site in human compared to chimp. The human:(chimp-mouse) ratio is the estimated number of substitutions per site per million years in human compared to the chimp-mouse phylogeny. Note that because rodent generation times are much shorter than human, we expect the rates in years to be *smaller* in human than in the chimp-mouse if the regions are evolving at the same rate per generation. Branch lengths for scaling rates in millions of years: human=5my, chimp-mouse=150my. Totals computed by concatenating the five elements.
