# Supplementary material for: Forces Shaping the Fastest Evolving Regions in the Human Genome
Source: PLoS Genet. 2006 Oct 13;2(10):e168. doi: 10.1371/journal.pgen.0020168 (PMC1599772; doi:10.1371/journal.pgen.0020168)
Supplement: Supplementary file 14 [file pgen.0020168.st006.pdf]

	HAR1	HAR2	HAR3	HAR4	HAR5
4d sites					
**All**	$3.3e^{-14}$	$4.7e^{-11}$	$8.3e^{-5}$	$1.2e^{-3}$	$5.5e^{-4}$
**Last Band**	$2.4e^{-12}$	$1.8e^{-9}$	$4.7e^{-4}$	$4.9e^{-3}$	$4.8e^{-3}$

Table S6. Significance of human acceleration. Wright-Fisher independent sites model p-values for human substitutions compared to the genome-wide human-chimp background rate based on ENCODE region[39] 4d sites and the human background rate in 4d sites of ENCODE regions in final chromosome bands. Unadjusted p-values are reported.
