# Supplementary material for: Forces Shaping the Fastest Evolving Regions in the Human Genome
Source: PLoS Genet. 2006 Oct 13;2(10):e168. doi: 10.1371/journal.pgen.0020168 (PMC1599772; doi:10.1371/journal.pgen.0020168)
Supplement: Supplementary file 15 [file pgen.0020168.st007.pdf]

	HAR1	HAR2	HAR3	HAR4	HAR5
Index of dispersion					
**unweighted**	2.38	3.60	1.91	3.32	22.08
***p*-value**	0.024	0.0063	0.057	0.14	0.89
**weighted**	11.28	13.75	6.95	5.90	8.26
***p*-value**	0.087	0.024	0.14	0.029	0.00

Table S7. Index of dispersion in HAR1-HAR5. Weighted index of dispersion computed using the method of Gillespie[27] P-values computed by simulation (not adjusted for multiple comparisons).
