# Supplementary material for: Forces Shaping the Fastest Evolving Regions in the Human Genome
Source: PLoS Genet. 2006 Oct 13;2(10):e168. doi: 10.1371/journal.pgen.0020168 (PMC1599772; doi:10.1371/journal.pgen.0020168)
Supplement: Supplementary file 16 [file pgen.0020168.st008.pdf]

		HAR1	HAR2	HAR3	HAR4	HAR5
Standard neutral						
**1Kb**	n=10	0.01981	0.08308	0.75312	0.99231	0.72593
	n=50	0.00433	0.06367	0.77749	0.97381	0.73229
	n=100	0.00241	0.06628	0.80073	0.96101	0.73589
**5Kb**	n=10	0.04668	0.00491	0.66538	1.00000	0.49262
	n=50	0.00475	0.00035	0.71500	1.00000	0.48233
	n=100	0.00152	0.00015	0.74426	1.00000	0.48350
**10Kb**	n=10	0.49164	0.39595	0.16318	1.00000	0.67670
	n=50	0.45388	0.34431	0.06142	1.00000	0.72524
	n=100	0.42251	0.36917	0.05721	1.00000	0.74191
Recent expansion						
**1Kb**	n=10	0.00656	0.06680	0.72848	0.61229	0.72620
	n=50	0.00073	0.06078	0.82650	0.43900	0.80345
	n=100	0.00112	0.05803	0.83592	0.34701	0.68332
**5Kb**	n=10	0.01105	0.00214	0.63092	0.95413	0.52108
	n=50	0.01060	0.00017	0.76901	0.75883	0.61696
	n=100	0.00037	0.00011	0.78050	0.55511	0.61425
**10Kb**	n=10	0.26667	0.34493	0.12145	0.84908	0.68131
	n=50	0.22139	0.37124	0.06795	0.38839	0.82961
	n=100	0.38992	0.34466	0.04269	0.14957	0.84986
Ancient expansion						
**1Kb**	n=10	0.02351	0.08975	0.84984	0.77039	0.63095
	n=50	0.00307	0.06582	0.87187	0.54836	0.75420
	n=100	0.00131	0.06184	0.87523	0.51468	0.77742
**5Kb**	n=10	0.03977	0.00189	0.80463	0.99711	0.36589
	n=50	0.00183	0.00008	0.84652	0.96960	0.48632
	n=100	0.00015	0.00006	0.72705	0.87804	0.52350
**10Kb**	n=10	0.65032	0.50698	0.19286	0.99066	0.53724
	n=50	0.64041	0.42026	0.01294	0.71901	0.75664
	n=100	0.52263	0.39300	0.01083	0.62670	0.81869
Bottleneck						
**1Kb**	n=10	0.03071	0.05847	0.86146	0.27896	0.83247
	n=50	0.00135	0.06354	0.89160	0.29777	0.85703
	n=100	0.00106	0.04441	0.83491	0.24584	0.80315
**5Kb**	n=10	0.03936	0.00003	0.83600	0.37603	0.65098
	n=50	0.00005	0.00002	0.89848	0.42068	0.71374
	n=100	0.00007	<0.00001	0.78958	0.27764	0.57119
**10Kb**	n=10	0.97675	0.34636	0.03201	0.07156	0.90889
	n=50	0.67266	0.39456	0.02863	0.07313	0.95952
	n=100	0.68356	0.22591	0.00668	0.02365	0.88843

Table S8. Selective sweep test p-values. Significance of the observed pattern of polymorphism and divergence for each HAR compared to the surrounding 1Mb. Coalescent-based simulations were performed with four demographic models: the standard neutral model, a model with a recent population expansion, a model with an ancient population expansion, and a model with a population bottleneck followed by expansion. For each HAR, a separate analysis was conducted under each model at each combination of three scales (windows of size 1Kb, 5Kb, and 10Kb) and three sample sizes ($n = 10,50,100$) as described in Text S1.10. The p-values are the proportion of the 10^5 simulated data sets that have the observed number or fewer segregating sites, conditional on the observed human-chimp divergence and the speciation time estimated from the surrounding 1Mb regions. Unadjusted p-values are reported.
