# Supplementary material for: Forces Shaping the Fastest Evolving Regions in the Human Genome
Source: PLoS Genet. 2006 Oct 13;2(10):e168. doi: 10.1371/journal.pgen.0020168 (PMC1599772; doi:10.1371/journal.pgen.0020168)
Supplement: Supplementary file 17 [file pgen.0020168.st009.pdf]

	HAR1	HAR2	HAR3	HAR4	HAR5
**Chromosome**	chr20	chr2	chr7	chr16	chr12
**Start**	61,203,891	23,655,6008	1,979,165	71,686,758	844,433
**Length**	187 bp	155 bp	295 bp	463 bp	418 bp
Human Differences					
**W→S**	23	11	10	6	4
**S→W**	0	0	0	3	1
**neither**	2	1	1	2	3
**Human:Chimp**	12.97	23.08	26.62	37.53	12.79

Table S9. Details of phastCons elements containing HAR1-HAR5. Coordinates are from hg17 human genome assembly (build 35). Human differences gives the number of bases that do not match the human-chimp-macaque consensus ancestral sequence. The human:chimp ratio is the estimated substitutions rate per million years in human compared to chimp. Rates are estimated using the method described in Siepel *et al*[38]. with the CONS model as prior (Text S1).
