# Supplementary material for: Forces Shaping the Fastest Evolving Regions in the Human Genome
Source: PLoS Genet. 2006 Oct 13;2(10):e168. doi: 10.1371/journal.pgen.0020168 (PMC1599772; doi:10.1371/journal.pgen.0020168)
Supplement: Supplementary file 18 [file pgen.0020168.st010.pdf]

Name	Forward primer	Reverse primer	Size (bp)	Primates amplified
HAR1	gctgacagggttcgaaagag	ttgctgtcctgcttcttcct	429	H
HAR1	ccctgttctcccctcttttc	agagaccctgagtcgcagtc	280	C,G,O
HAR1	tgtaatcagccgctaagacg	caattaaaatccccgtgctg	275	M
HAR2	agccagacctaatgtgaacc	ggccgattctggatctttaa	666	H,C,G,O,M,S
HAR3	tggtccatccagacctcg	gcctgcttgtgtgtggttg	721	H,C,O,M
HAR4	ctgggctaattaagcagactgag	agttgcccggtatcaatcct	398	H,C,G,O,M,S,L
HAR5	tgtcagatgtgctatgttgag	tgtgccaaggacattatagg	652	H,C,G,O,M

Table S10. Resequencing primers. Details of the primers used in this study. H=human, C=chimpanzee, M=macaque, G=gorilla, O=orangutan, S=spider monkey, L=lemur.
