# Supplementary material for: Forces Shaping the Fastest Evolving Regions in the Human Genome
Source: PLoS Genet. 2006 Oct 13;2(10):e168. doi: 10.1371/journal.pgen.0020168 (PMC1599772; doi:10.1371/journal.pgen.0020168)
Supplement: Supplementary file 19 [file pgen.0020168.st011.pdf]

	Human Diffs	Low Quality Human Diffs	Additional Polymorphisms
**HAR1**	10/10	2/2	0
**HAR2**	8/8	4/4	0
**HAR3**	5/5	1/1	0
**HAR4**	5/5	0/0	0
**HAR5**	7/8	0/0	2

Table S11. PDR panel sequencing results. Resequencing of the 24-member subset of the NHGRI Polymorphism Discovery Resource Panel[41]. Nearly all human-specific substitutions are fixed in the panel, including all low quality human diffs (Text S1.1).
